# Ontology- and Data-Driven Circularity Assessment of Waste-Derived Rigid PVC-u: A Micro–Macro Decision Framework for Sustainable Recycling and Smart Manufacturing

**DOI:** 10.3390/polym18202525

**Published:** 2026-10-15

**Authors:** Alexander Chinaka Chidara, Shouxun Ji, Anietie Austin Awak

**Affiliations:** 1 Department of Mechanical and Aerospace Engineering, College of Engineering, Design and Physical Sciences, Brunel University of London, Uxbridge UB8 3PH, UK; 2 Brunel Centre for Advanced Solidification Technology (BCAST), Brunel University of London, Uxbridge UB8 3PH, UK; 3 School of Business and Strategic Management, Yaba College of Technology, Lagos 100252, Nigeria; anietie.awak@gmail.com

**Keywords:** waste-derived rigid PVC-u, circularity assessment, ontology, Protégé, big data analytics, circular economy, smart manufacturing, sustainable recycling, micro- and macro-level assessment, SWRL, SPARQL

## Abstract

The circularity of waste-derived rigid unplasticised poly (vinyl chloride) (PVC-u) is constrained by heterogeneous waste-stream sources, degraded material quality, legacy additives, and inconsistent decision criteria spanning technical, environmental, and economic aspects. In this work, we developed an ontology- and data-driven circularity assessment framework for waste-derived rigid PVC-u recycling systems, built on a Protégé ontology coupled with big data analytics. The framework structures knowledge on materials, recycling processes, process parameters, quality aspects, sustainability measures, carbon aspects, and cost aspects, and links it to process, operational, and business data. Semantic reasoning, based on rule-based and query-based logics, derives circularity, environmental, and economic measures and improves assessment consistency across different recycling cases. The assessment is carried out from two interconnected perspectives: a micro-level perspective focused on efficiency, quality, waste, energy, cost, and emissions at the plant level, and a macro-level perspective focused on circularity, resource retention, industrial value recovery, and net-zero implications at the system level. The combination of ontological modelling and analytics-driven assessment facilitates transparent, rule-based and traceable circularity assessment of recycling, processing and value recovery pathways for waste-derived rigid PVC-u. Applying the framework to three illustrative, literature-informed synthetic scenarios illustrates its ability to translate plant-level data into consistent, traceable micro- and macro-level circularity assessments aligned with circular economy and net-zero goals. Monte Carlo propagation of the scenario parameter ranges shows that every recycling route remains a net emission saver, whereas cost recovery and carbon-price viability differ strongly between routes, and that the 2030 circularity targets are met only if about 60% (circular material use) to 81% (end-of-life recycling) of the projected improvement is realised. These results are illustrative: we have not yet used industrial plant data, and empirical validation with recyclers is a priority for future work. This paper contributes an ontology- and data-driven circularity assessment framework for the sustainable recycling and smart manufacturing of waste-derived rigid PVC-u and outlines an approach to combine semantic intelligence with industrial analytics for circularity assessment.

## 1. Introduction

### 1.1. Background to Rigid PVC-u Recycling and Circular Manufacturing

Poly (vinyl chloride) (PVC) ranks as the third most commonly produced synthetic polymer, with annual production exceeding 40 million tonnes [1]. Among PVC types, unplasticised PVC (PVC-u) makes up a sizeable share of rigid applications, including pipes, window profiles, siding, and construction materials [2]. The rigidity, chemical resistance, and mechanical strength of rigid PVC-u have been indispensable in infrastructure and construction applications, but these features pose major challenges for end-of-life management [3].

Moving away from the traditional take-make-dispose approach towards more circular manufacturing models has spurred interest in polymer recycling [4,5]. According to circular economy principles, materials should retain value across multiple life cycles, with recycling as an essential part of this process [6,7]. For rigid PVC-u, circularity is further complicated by legacy additives such as lead-based stabilisers, phthalate plasticisers (though rare in rigid PVC-u), and various fillers [8,9,10].

This study focuses on rigid PVC-u for three reasons. First, it is used mainly in long-life construction products such as pipes, window profiles and cladding, so large and geographically concentrated volumes are now reaching end of life. The sector has committed to recycling 1 Mt of PVC per year by 2030 [11], and the revised Construction Products Regulation introduces environmental sustainability and digital product information requirements [12]. Second, rigid PVC-u streams are more homogeneous than plasticised streams (cables, flooring, films), which carry high and variable plasticiser contents. A standard for controlled loop recycling already exists for window and door profiles [13]. Third, rigid PVC-u carries the legacy lead problem described in Section 1.2. This makes decision support, and not only technology, the binding constraint, in line with system-level analyses of PVC circularity in the built environment [14].

Flexible PVC is deliberately outside the present scope. Rigid streams may still contain fragments of plasticised PVC such as gaskets and seals. Because plasticiser content lowers density, sink-float and spectroscopic sorting are the usual upstream responses, and the sequence of sorting steps strongly affects operating cost [15]. The ontology does not yet represent plasticised material explicitly, so its outputs should not be relied upon for feedstock with a substantial plasticised fraction. Extending the framework to mixed rigid-flexible feedstocks would require plasticiser-specific classes and migration and compatibility rules (Section 12.5). Whether recycling facilities would adopt such a tool has not been assessed empirically. The drivers addressed here (compliance with lead limits, recycled-content and end-of-life declarations, and carbon reporting) are regulatory and market requirements, and stakeholder acceptance will be tested in the planned industrial case studies.

### 1.2. Sustainability Challenges in Rigid PVC-u Recycling Systems

Recycling rigid PVC-u faces several interrelated issues. First, heterogeneous waste streams from the construction and demolition industries and municipalities, with different PVC-u types, contamination, and additive content [8,16], pose a challenge. Second, thermal degradation during mechanical recycling causes dehydrochlorination, leading to discolouration, reduced mechanical properties, and the release of corrosive hydrogen chloride [17,18,19]. Third, legacy additives, especially those banned or controlled under REACH and the Stockholm Convention, hinder closed-loop recycling and may cause downstream contamination [9,10].

Lead is the clearest case. Lead stabilisers were phased out of virgin PVC production in Europe by 2015, but lead-stabilised products installed earlier remain in service for decades and continue to reach end of life. In Germany, lead concentrations in end-of-life window-profile waste are projected to stay above 0.3% until the end of this century without restriction [20]. Regulation (EU) 2023/923 [21] amends Annex XVII of the REACH Regulation [22] and now limits lead in PVC articles to below 0.1 wt%. A derogation applies until 28 May 2033 for specified rigid articles made from recovered rigid PVC containing less than 1.5 wt% lead, subject to labelling.

Lead therefore remains a live decision variable for recyclers even though it is banned in new production, which is why lead-bearing feedstock is modelled in the scenarios of Section 9.

Chemical recycling methods such as pyrolysis and solvent-based dissolution are promising ways to valorise PVC-u waste, but they face many technical and economic challenges [23,24,25]. The efficiency of the dechlorination process, energy requirements, and product variability are among the problems that have to be overcome [19,26,27]. Although De Meester et al. focus on WEEE rather than PVC-u, they highlight a similar lack of integrated sustainability assessment methodologies for decision-making that is also evident in PVC-u recycling research [28,29].

### 1.3. Micro- and Macro-Sustainability Decision Gaps

Recycling system sustainability decisions span several scales of operation and are rarely considered collectively. Micro-sustainability decisions consider operational characteristics at the plant level, such as energy per unit processed, yields, waste levels, costs, and quality of products [30,31]. Macro-sustainability decisions consider the system-level impacts such as circularity metrics, resource retention efficiency, value recovered throughout the supply chain, net-zero contribution, and policy compliance [32,33].

Existing decision-support technologies fail to adequately combine these two scales. LCA provides comprehensive environmental impact assessment, but its results are not linked to operational data [34,35]. MFA assesses resource flows, but gives little guidance for decisions [36,37]. Economic calculators describe cost structures, but do not include environmental externalities [38,39]. The missing link is an approach that combines micro-level operational information with macro-level sustainability goals using a well-defined knowledge representation framework.

### 1.4. Role of Ontology, Big Data Analytics and Smart Manufacturing Economics

Ontology engineering provides methods for systematically formalising knowledge in complex domains [40,41]. In sustainability studies, researchers have developed ontologies for manufacturing processes, eco-design, and materials science to ensure semantic interoperability and reasoning across heterogeneous knowledge sources [42,43,44]. Protégé is an ontology authoring tool that facilitates ontology creation, visualisation, and querying [45,46].

Big data analytics has transformed manufacturing intelligence through pattern recognition, anomaly detection and predictive modelling of process and operational data streams [47,48,49]. Industry 4.0 concepts focus on data-driven decision-making using sensors, cyber-physical systems, and digital twins [50,51]. When combined with formal ontologies, big data analytics can support semantic reasoning that draws on both structured domain knowledge and industrial data [52,53].

### 1.5. Research Aim, Objectives and Research Questions

Aim: To develop an ontology- and data-driven circularity assessment framework that evaluates and supports micro- and macro-level decisions for the sustainable recycling and smart manufacturing of waste-derived rigid PVC-u.

Research Objectives: (1) To develop a Protégé-based ontology that formalises the materials, processes, quality parameters, sustainability metrics and economic factors relevant to circularity assessment of waste-derived rigid PVC-u. (2) To integrate process-related, operational and business data into an ontology-based circularity assessment environment. (3) To apply semantic reasoning and analytics for the calculation and interpretation of environmental, carbon, cost and circularity indicators within waste-derived rigid PVC-u recycling scenarios. (4) To evaluate the effectiveness of the proposed circularity assessment framework in supporting recycling and smart manufacturing decisions at micro- and macro-levels.

Research Questions: How can a Protégé-based ontology represent the relationships that determine the circularity of waste-derived rigid PVC-u recycling systems? How can big data analytics be integrated with ontology modelling to support circularity assessment of waste-derived rigid PVC-u recycling systems? How can environmental, carbon, cost and circularity indicators be computed and interpreted within a micro-macro circularity assessment framework? How can the resulting circularity assessment framework improve sustainable recycling and smart manufacturing decisions for waste-derived rigid PVC-u?

Scope and evidence base. The framework is demonstrated using synthetic, literature-informed scenario data, not measurements from an operating recycling plant. The results in Section 9 therefore illustrate how the framework processes information, along with an uncertainty analysis of what those illustrative inputs imply. They are not empirical performance estimates, and the limitations in Section 11 should be read alongside every quantitative statement.

### 1.6. Structure of the Paper

The paper’s outline is given as follows: Section 2 provides a critical literature review. Section 3 introduces the conceptual framework that links ontology, big data analytics, and decision-making for circularity assessment. Section 4 describes the methodology, including ontology building, data needs, and analytical approaches. Section 5 and Section 6 provide the ontology model and framework for big data analytics, respectively. Section 7 and Section 8 discuss the micro- and macro-level circularity decision-making frameworks. Section 9 demonstrates the framework through illustrative scenarios and reports uncertainty, sensitivity and robustness analyses. Section 10 discusses contributions and implications; Section 11 sets out the limitations; Section 12 presents the conclusions, policy recommendations, and future research.

## 2. Literature Review

### 2.1. Rigid PVC-u Recycling Systems and Circular Economy Principles

The circular economy has reframed plastics as a stock whose value should be retained rather than a waste stream to be managed [32,54,55], yet critical analyses caution that thermodynamic limits, quality loss and rebound effects constrain how far material loops can be closed in practice [56]. Rigid PVC-u illustrates this tension. Mechanical recycling has been an established industrial practice for more than three decades [17,25], and Europe recycled 737,645 t of PVC in 2023, against a sector commitment of 1 Mt per year by 2030 [11]. However, thermo-mechanical action shortens polymer chains and erodes properties with each reprocessing step [57]. Laboratory work on stabilised rigid PVC suggests that up to four reprocessing cycles do not impair impact strength or heat resistance [58], but these studies use well-characterised compounds rather than decades-old post-consumer material with an unknown formulation. They therefore bound, rather than predict, real quality loss, which is why the present framework treats quality as an assessed input and not as an assumed constant.

### 2.2. Material, Additive, Contamination and Quality Challenges

Additives are the central obstacle to closing PVC loops. Hahladakis et al. [8] and Wiesinger et al. [10] show that the chemical space of plastic formulations is very large and that its complexity hinders recycling, while Wagner and Schlummer [9] demonstrate that substances banned from production persist in recycled products; Schlummer et al. [59] make the same point for polymer fractions from electronic waste. Lead is the clearest example for rigid PVC-u: it left virgin production by 2015, yet lead concentrations in end-of-life window profiles are projected to remain above 0.3% for decades [20], and Regulation (EU) 2023/923 now caps lead in PVC articles at 0.1 wt% with a time-limited derogation for recovered rigid PVC below 1.5 wt% [21]. Legacy lead is thus a live decision variable rather than a historical footnote. Recyclate quality is normally judged by intrinsic viscosity, colour stability, filler content and contaminants [29,60,61], and EN 17410 defines controlled-loop recycling for window and door profiles [13]. The literature lacks a machine-readable link between these quality parameters, regulatory thresholds, and route selection.

### 2.3. Chemical Feedstock Recycling and Pyrolysis

Chemical recycling is usually presented as a complement to mechanical recycling for streams that are too contaminated or degraded [23,24,62]. For PVC, dechlorination is the gating step, whether achieved by fluidised-bed pyrolysis with hydrogen chloride recovery [63], by in situ or pre-treatment dechlorination of pyrolysis fuels [64], by microwave heating at lower energy demand [26] or by non-thermal plasma [27]; systematic reviews confirm that dechlorination efficiency, energy demand and product variability remain unresolved [19]. Recent techno-economic and life cycle assessment of chemical recycling of mixed plastics containing PVC shows that the choice of dechlorination route affects both process economics and emissions [65]. Most studies report laboratory-scale kinetics or product yields and rarely link them to plant-level costs or life-cycle consequences, limiting their use for routing decisions.

### 2.4. Solvent-Based and Dissolution Recycling

Solvent-based (dissolution and precipitation) routes separate polymer from additives and contaminants without thermally damaging the polymer [66,67]. For rigid PVC, Aerts et al. [68] recovered PVC with titanium dioxide and calcium carbonate using bio-based solvents, and Pappa et al. [69] demonstrated selective dissolution at pilot scale without losing polymer properties. Reviews stress that solvent choice, recovery efficiency and energy demand govern environmental performance [66], and Ügdüler et al. [67] offer selection criteria, whereas approaches such as solvent-targeted recovery and precipitation were developed for multilayer packaging rather than PVC [70]. Solvent routes are therefore technically credible for additive-laden rigid PVC, but their economic and carbon trade-offs against mechanical routes are seldom quantified consistently.

### 2.5. Life Cycle Assessment, Carbon Accounting and Circularity Metrics

Life cycle assessment is the standard tool for comparing recycling routes [71,72], but results depend on allocation, avoided-burden and temporal choices [34], on impact methods and inventories [73,74], and on system-boundary and end-of-life modelling for recyclable plastics [39]. Pathway studies indicate that chemical recycling combined with carbon capture and utilisation can cut greenhouse-gas emissions substantially [75], and comparisons of closed-loop technologies for common plastics assess technical, economic and environmental criteria together [76].

PVC-specific comparisons linked to a reusable semantic representation remain absent. Circularity metrics such as the product-level indicator of Linder et al. [33] and the Ellen MacArthur Foundation Material Circularity Indicator [32] complement life cycle assessment, but they were not designed for quality-graded, multi-route decisions, and policy mixes determine whether recycling outcomes materialise [77].

### 2.6. Micro- and Macro-Sustainability Indicators

Micro-level indicators (energy per kilogram, waste generation, material efficiency, emissions intensity) serve plant managers [30,31,72], whereas macro-level indicators (circular material use rate, end-of-life recycling rate, resource efficiency and avoided emissions) serve system planners [32,33,77]. The two are usually computed in separate tools by separate stakeholders. Zink et al. [78] show that macro-level avoided-production credits depend on market-based substitution assumptions, and Velenturf and Purnell [79] argue that sustainable circular-economy principles require explicit system boundaries. The unresolved problem is traceable aggregation: how plant-level data become system-level indicators without opaque recalculation.

### 2.7. Ontology Modelling and Protégé-Based Reasoning

Ontologies formalise shared conceptualisations [40,41] and, expressed in OWL [80] and RDF [81], provide standard, reasoner-compatible representations, with SWRL rules [82] and SPARQL queries [83] supporting inference and retrieval. Sustainability ontologies exist for manufacturing [84], including frameworks that link shop-floor data to environmental performance indicators [44] and materials ontologies for data interoperability [42,43], and rule-based ontologies have been used for process quality analysis [85]. The closest work to the present study is an ontology-driven semantic simulation of sustainable manufacturing for PVC composites [86], the authors’ earlier ontology-based modelling of sustainable polymer systems, which was tested in a UK industrial case study that identified additive complexity as a major barrier to PVC recycling [87], and a material-passport ontology for the circular economy [88]. The authors’ recent illustrative ontology for PVC materials combines lifecycle simulation, literature analytics, and Protégé reasoning, but is not a validated decision-support tool [89]; digital product passports have also been proposed as a data source for deriving circular strategies [90]. These works show that ontology-based reasoning is feasible in this domain, but none couples rigid PVC-u route selection with plant-level analytics, economics and macro-level circularity indicators.

### 2.8. Big Data Analytics for Circular Smart Manufacturing

Big data analytics, characterised by volume, velocity, variety, veracity and value [47,49], supports predictive maintenance, quality management and energy management in manufacturing [50,51,91], and digital twins synchronise virtual replicas with real-time data [91,92]. In PVC processing, a data-driven digital twin built from real extrusion-line data for rapid start-up shows that machine learning on plant data is practicable [93]. In circular-economy contexts, analytics have been linked to supply-chain flexibility and sustainable performance [94] and artificial intelligence to plastic recycling and upcycling strategies [95], with deep learning applied to polymer-property prediction [96,97]. Much of this work optimises single processes and rarely propagates results to circularity or net-zero indicators.

### 2.9. Research Gap and Conceptual Positioning

Taken together, the literature offers strong but separate contributions on PVC recycling technology, life-cycle and circularity assessment, ontology engineering and industrial analytics. What is missing is an integrated framework that (i) represents rigid PVC-u materials, additives, processes and quality in a machine-readable form, (ii) ties regulatory thresholds and plant data to route decisions, (iii) aggregates micro-level results into macro-level circularity and net-zero indicators, and (iv) exposes the economic and uncertainty implications of those decisions. The proposed framework addresses this gap; as Section 9 and Section 11 make clear, it does so at the level of a demonstrated concept rather than a plant-validated tool.

## 3. Conceptual Framework Development

### 3.1. Framework Logic and Theoretical Foundation

This conceptual framework is founded upon three distinct theoretical perspectives: knowledge-based decision theory [41], according to which better decisions are made through knowledge structure, accessibility, and reusability in the domain; multiple criteria decision analysis [98], which considers that decision-making in recycling involves compromising between several goals such as technical, environmental and economic ones; and circular economy systems thinking [7].

Table 1 presents the framework’s core logical components and their theoretical foundations.

### 3.2. Micro-Sustainability Decision Layer

The micro-level circularity decision-making layer focuses on decision-making regarding plant-level operations. These include feedstock acceptance (impurity, additive composition, moisture content), process parameters (temperature, residence time, screw speed), quality control (melt flow index, colour, mechanical properties), maintenance schedule (throughput, energy consumption, wear indicators), and product distribution (requirement for secondary applications). The micro-layer works on data streams such as sensor data (temperature, pressure, energy consumption), lab results (mechanical properties, colour, molecular weight), and operational data (downtime, cleaning interval, yield).

### 3.3. Macro-Sustainability Decision Layer

Macro-sustainability decisions involve the system-level considerations of technology investment (capital expenditure, operating expenditure, environmental gain); circular economy targets (recycling target, recycled content, residual material handling); compliance with policy (policy mandates, reporting); supply chain structure (collection process, recycling facility, end-market); and net-zero strategy (cost of carbon reduction, technological readiness). The macro-decision level integrates data from multiple micro-decision levels within the market, policy, and environmental context.

### 3.4. Circular Smart Manufacturing Economics Layer

The economics dimension measures value generation and appropriation within the model. Economic variables consist of cost items (collection, sorting, washing, shredding, recycling and quality control), non-cost items (overheads, administration, compliance, depreciation), value recovery (revenue from secondary products in €/tonne, by-product valorisation) and externalities valued (price of carbon in €/t CO_2_e, waste disposal savings, value of virgin material substitution).

**Figure 1:**
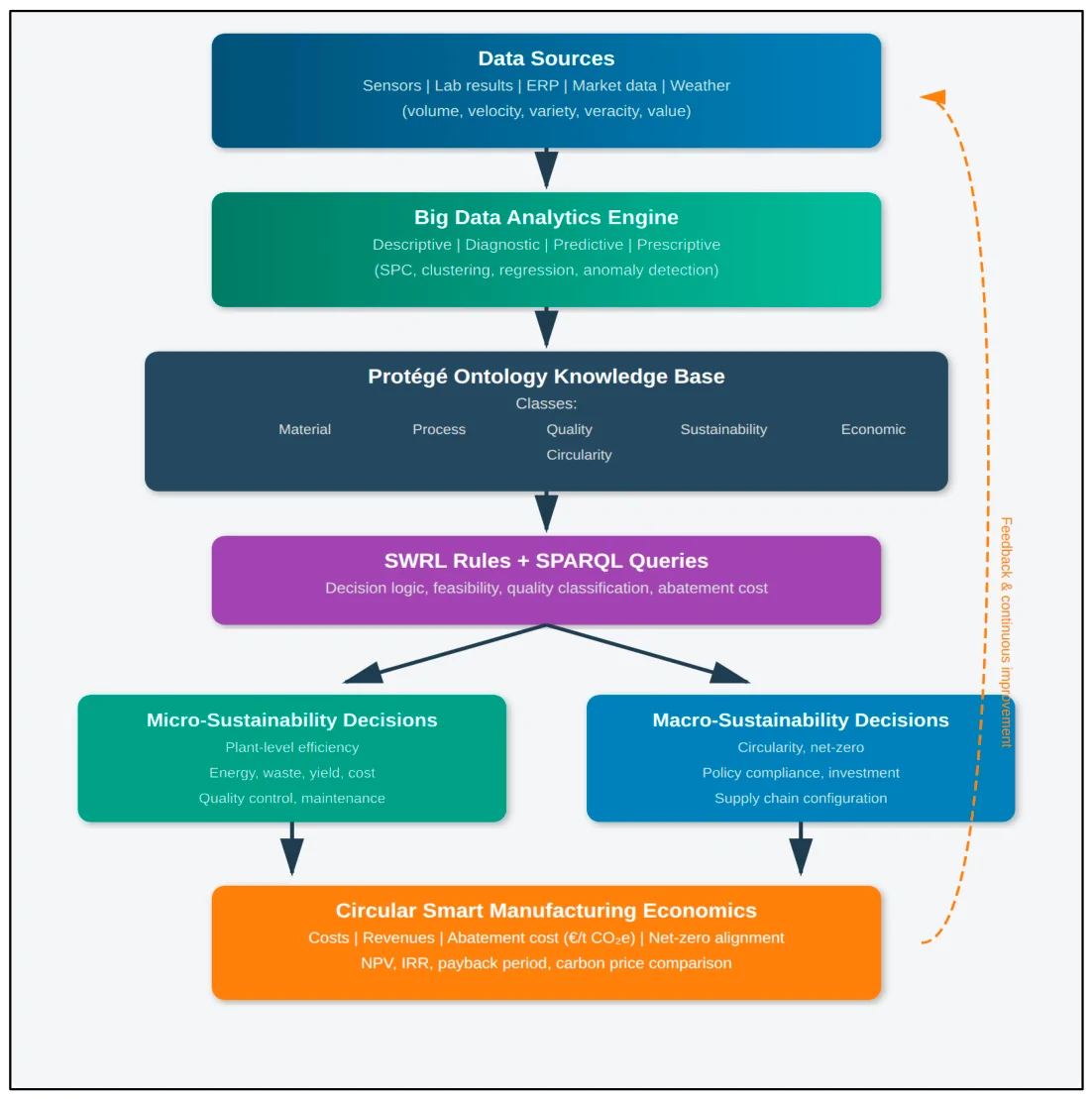
Conceptual architecture of the ontology- and data-driven circularity assessment framework for waste-derived rigid PVC-u (author’s own elaboration).

Figure 1 illustrates the conceptual framework architecture, with layers representing the ontology knowledge base, big data analytics engine, micro- and macro-decision modules, and the circular smart manufacturing economics integrator.

### 3.5. Ontology-Enabled Decision-Support Architecture

The ontology plays an important role as the semantic glue that binds the framework pieces together. The basic ontologies include: the material ontology (grades of PVC-u, K-value, molecular weight distribution, fillers like CaCO_3_ and TiO_2_, additives such as stabilisers, lubricants and processing aids), the process ontology (recycling process options encompassing mechanical, chemical and solvent-based recycling, process steps like shredding, washing, separation and extrusion, process parameters like temperature and residence time), the quality ontology (property groups such as mechanical, thermal, rheological and optical properties, measuring techniques following ISO/ASTM standards, specification limits), the sustainability ontology (categories of impacts such as global warming potential, ecotoxicity and resource depletion, indicator definitions, normalisation criteria), the economic ontology (cost types, value chains, investment performance measures like NPV, IRR and payback period), and finally the decision ontology (micro-, macro- and strategic decision scenarios linking the sustainability indicators, which may include circular economy metrics like loop type, retention methods and closed/open-loop classification, to action recommendations).

### 3.6. Big Data Analytics Integration Pathway

Big data analytics integrates with ontology through data annotation (tagging datasets with ontology class and property URIs), semantic federation (querying distributed data sources using ontology-aligned SPARQL), analytics triggering (ontology rules invoking analytic workflows when conditions are satisfied) and result interpretation (mapping analytic outputs back into ontology instances for reasoning).

## 4. Methodology

### 4.1. Research Design

This research employs a design science methodology suitable for artefact-based research [99]. The research is conducted in five stages, namely identification of decision gaps in the sustainability of rigid PVC-u recycling (Section 1 and Section 2); definition of solution objectives (Section 3); requirement specification for the ontology and analytics framework; artefact design and development (Section 4, Section 5, Section 6, Section 7 and Section 8), ontology and analytics framework creation based on the Protégé platform; demonstration (Section 9) showing the use of the ontology and analytics framework through case scenarios; and evaluation (Section 10), assessing framework utility against defined objectives. The ontology was developed using Protégé Desktop (version 5.6.9; Protégé project, Stanford University, Stanford, CA, USA) [100].

### 4.2. Data Requirements and Data Sources

Data requirements differ depending on the framework components. The material ontology data, such as PVC grades, additives, and fillers, are obtained from literature [1,2,3,8]. Process ontology data, including unit operation parameters and boundaries, are acquired from literature [6,17,25] and technical standards. Data for the quality ontology include the property range, test method, and specification limits from ISO standards and the literature [29,60]. Characterisation factors and normalisation references for the sustainability ontology data are from Ecoinvent [74] and ReCiPe [73]. Economic ontology data include cost structure and market prices from industry reports and literature [38,39].

Process, quality and energy data required for analytics integration are synthetic: they were constructed from the literature ranges above and are not measurements from an operating plant, because this is a framework development study and not a primary industrial data collection study. Each input used in Section 6, Section 7, Section 8 and Section 9 therefore belongs to one of three provenance classes: (i) literature- or standard-derived values (for example, the PVC-u grade properties of Section 5.2 and regulatory limits), (ii) literature-informed illustrative ranges (Section 5.6, Section 6.4 and Section 8.3), and (iii) purely illustrative scenario values (the scenarios of Section 9.2, Section 9.3, Section 9.4 and Section 9.5). Section 9.6 records the provenance of the main decision thresholds. Because the demonstration data are synthetic and noise-free, we examine robustness to measurement noise separately in Section 9.6.

### 4.3. Protégé Ontology Development Method

We developed the ontology according to the methodology proposed by Noy and McGuinness [101]. The first stage established the domain and scope by restricting it to rigid PVC-u recycling systems and the sustainability aspects of decision-making. The second stage evaluated the possibility of reusing existing ontologies, such as the PMD Core Ontology [42] and materials ontologies [43], as a base, but further extension was needed to fit PVC-specific terms. At the third stage, important terms were identified; they include materials (different PVC-u grades, additives, fillers, contaminants), processes (collecting, sorting, cleaning, shredding, separation, reprocessing), quality characteristics (K-value, colour, mechanical characteristics, thermal stability), sustainability indicators (carbon footprint, energy intensity, circularity parameters) and economic parameters (costs, revenues, investments). The next step was to identify the class and hierarchy structure based on six top-level classes (Material, Process, Quality, Sustainability, Economic, Decision_Context). Then we defined the properties, including object properties (relations between individuals of different classes) and datatype properties (relations between individuals and literal values). The sixth stage defined constraints, including cardinality and domain-range constraints. The final stage instantiated some classes, PVC-u grades (e.g., PVC-u K65, K67, K70), types of stabilisers (Ca/Zn, organotin, lead), and recycling processes.

### 4.4. Ontology Classes, Properties and Relationships

Table 2 presents the primary ontology classes and subclasses.

Key object properties include has_material_composition (PVC_u_Product → Material_Component), is_processed_by (PVC_u_Waste → Recycling_Process), exhibits_quality (Recyclate → Quality_Attribute), impacts_sustainability (Recycling_Process → Sustainability_Indicator), incurs_cost (Recycling_Process → Cost) and supports_decision (Sustainability_Indicator → Decision_Context).

### 4.5. SWRL Rule and SPARQL Query Design

SWRL rules encode decision logic for sustainability assessment. Example rules include a recyclate quality classification rule: PVC_u_Waste (?w) ∧ has_contaminant_level (?w, ?cl) ∧ swrlb:greaterThan (?cl, 0.05) → has_quality_grade (?w, “Low_Grade”).

A mechanical recycling feasibility rule is: PVC_u_Waste (?w) ∧ has_additive_type (?w, “Pb_Stabiliser”) ∧ has_concentration (?w, ?conc) ∧ swrlb:greaterThan (?conc, 0.001) → requires_pretreatment (?w, “Lead_Management”). The threshold of 0.001 (0.1 wt%) corresponds to the limit for lead in PVC articles under Regulation (EU) 2023/923 [21]: feedstock between 0.1 and 1.5 wt% lead is routed either to the permitted rigid construction applications with the required labelling or to stabiliser-extraction pre-treatment, and feedstock above 1.5 wt% cannot be recycled into regulated articles without prior lead removal.

A micro-sustainability decision trigger rule is: Recycling_Process (?p) ∧ has_energy_intensity (?p, ?ei) ∧ swrlb:greaterThan (?ei, 800) → triggers_optimisation (?p, “Energy_Reduction”). Energy intensity is expressed in kWh/t, and the illustrative threshold of 800 kWh/t is the plant-level target of Section 7.1. Contaminant level and concentration are expressed as mass fractions (0.05 = 5 wt%). The provenance of these and other thresholds, and their sensitivity, are documented in Section 9.6.

SPARQL queries retrieve ontology instances for analytics. An example query retrieving all PVC-u grades with associated properties is: SELECT ?grade ?k_value ?density ?stabiliser_type WHERE {?grade rdf:type: PVC_u_Grade. ?grade: has_K_value ?k_value. ?grade: has_density ?density. ?grade: contains_additive ?additive. ?additive: has_type ?stabiliser_type}.

### 4.6. Big Data Analytics Approach

This analytics framework uses four types of analytics: descriptive analytics (aggregation and visualisation of sustainability metrics in recycling scenarios through summary statistics and dashboards), diagnostic analytics (detection of factor-result dependencies using correlation and feature importance assessment), predictive analytics (forecasting expected sustainability results in unmodelled scenarios via regression and machine learning algorithms), and prescriptive analytics (optimisation of decision variables to attain sustainability objectives under constraints). For this framework design research, the analytical methodologies are specified conceptually, based on applications in the literature [96,97,102].

### 4.7. Sustainability, Carbon, Cost and Circularity Indicator Selection

Table 3 presents the indicator framework.

Carbon factors follow IPCC [103] GWP100 characterisation factors. Cost data are expressed in 2024 euros. Circularity metrics follow the ISO 59020 [104] framework where applicable.

As regards system boundary and functional unit, all carbon and cost indicators are expressed per tonne of waste-derived rigid PVC-u accepted at the recycling facility gate (functional unit: 1 t of input; figures per tonne of output are stated explicitly where used). We apply two boundaries, consistent with ISO 14040 and ISO 14067 [30,72] and with Nordahl and Scown’s recommendations for recyclable plastics [39].

At the micro (plant) level, the boundary is gate-to-gate: inbound transport, sorting, cleaning, size reduction, the recycling process, post-processing, quality control and residue handling are inside, and cost indicators follow the same boundary.

At the macro (system) level, the boundary extends cradle-to-gate from the collection of end-of-life material to recyclate at the factory gate, with the disposal or treatment of residues inside the boundary. The burdens of the previous product life, the use phase, the conversion of recyclate into products, and the plant’s capital goods are excluded. The climate benefit of recycling is credited by system expansion: the emissions of the virgin PVC-u production that the recyclate displaces are avoided, and the credit is limited by the degree of substitution, which is why the avoided-emission ranges in Section 6.4 equal the full virgin baseline for closed-loop routes and are substantially lower for open-loop routes. Net emissions are thus process emissions minus avoided emissions, so a negative value denotes a net saving relative to virgin production. Plant-level (gate-to-gate) and system-level (cradle-to-gate with credit) indicators are not interchangeable and must not be summed.

### 4.8. Framework Validation Strategy

Validation is approached in three different ways: structural validation (evaluation of ontology completeness and consistency based on competency questions like “What recycling methods are applicable to PVC-u materials that contain lead stabilisers?”), operational validation (testing the analytics framework using sample problems obtained from the literature) and utility validation (critical evaluation of the results by an expert). These three forms of validation test internal consistency and plausibility only; they are not a substitute for validation against plant data, for which a protocol is set out in Section 11.

## 5. Protégé-Based Ontology Model for Rigid PVC-u Recycling

### 5.1. Ontology Scope and Domain Boundaries

The scope of the ontology encompasses decision-relevant entities and relationships for sustainable rigid PVC-u recycling. The scope excludes flexible PVC (plasticised) compounds that require their own stabilisation and processing considerations, PVC production from primary raw materials, and applications other than secondary product manufacture.

In line with the design-science artefact development method employed in Section 4, the quantifiable property value ranges, process parameters and instance values provided in Section 5, Section 6, Section 7, Section 8 and Section 9 are illustrative and representative of industry practice; specific values that are quoted from the relevant literature sources are identified, and others fill the ontology for the sake of demonstration.

The scope also excludes the recovery of lead from residue streams, such as stabiliser-extraction sludges, and from landfilled deposits: lead-bearing residues are represented only as an end-of-route residue stream (Section 5.7) and not as a recoverable resource.

### 5.2. Material and Recyclate Knowledge Representation

Material representation is characterised by differentiation of PVC-u resin (K-value K60–K70, polydispersity index 2.0–2.5, bulk density, particle size distribution), fillers (calcium carbonate 5–40% by weight, titanium dioxide 1–10% by weight, impact modifier like acrylic, chlorinated polyethylene), additives (heat stabiliser that includes lead, Ca/Zn, organotin, organic based, lubricants like paraffin wax, calcium stearate, PE wax, processing aid like acrylic copolymer), contaminants (polyolefin films, paper label, metal fragments, adhesive residues) and recyclate (post-industrial material which has a clean and known history compared to post-consumer material).

Table 4 lists PVC-u grades with typical property ranges.

### 5.3. Recycling Process and Route Representation

Mechanical recycling unit operations include shredding (rotary shear or hammer mill, 10–50 mm particle size, 50–200 kW power consumption), washing (cold or hot water, detergent addition at 0.1–1.0%, friction washer, 0.5–2.0 m^3^ water per tonne), separation (density separation by sink-float, electrostatic separation, colour sorting using NIR or hyperspectral imaging), agglomeration (frictional heat densification at 100–150 °C, 500–1500 rpm) and extrusion (twin-screw or single-screw, 180–210 °C, 50–500 kg/h throughput). The unit operations follow standard descriptions of mechanical recycling [17,25].

Chemical recycling representations include pyrolysis (400–600 °C, inert atmosphere, residence time 30–120 min, HCl recovery 85–99%), solvent-based dissolution (selective solvents such as MEK, THF or DMSO, 25–150 °C, polymer recovery 85–95%) and hydrothermal processing (250–400 °C, 5–25 MPa, alkaline conditions for dechlorination). Reviews of these routes are given in [62,66,67].

### 5.4. Quality, Contamination and Additive Variables

Quality attributes include mechanical properties (tensile strength 40–55 MPa, modulus 2500–3500 MPa, impact strength 2–8 kJ/m^2^ Charpy), thermal properties (Vicat softening temperature 75–85 °C, heat deflection temperature 70–80 °C), rheological properties (melt flow index 0.5–10 g/10 min at 190 °C/21.6 kg, melt viscosity) and optical properties (colour measured as ΔE* for discolouration, haze, gloss). Contaminant thresholds that trigger process adjustments are: polyolefin greater than 2%, requiring flotation separation; metal greater than 0.5%, requiring magnetic or eddy-current separation; paper greater than 1%, requiring hydrocyclone or additional washing; and adhesive residue greater than 0.1%, requiring solvent washing. These attributes follow common criteria for recyclate quality [29,60,61,109].

As regards reprocessing history and chain degradation, chain shortening and dehydrochlorination during reprocessing [17,18,57] are represented indirectly through measured quality attributes: K-value retention, melt flow index, colour change (ΔE*) and mechanical retention place the recyclate in a quality grade (Section 7.3), and no grade in Section 7.3 is mapped to pressure applications. The ontology does not yet record how many times a given material has been reprocessed, so cumulative degradation across successive life cycles is not modelled. Laboratory evidence for stabilised rigid PVC indicates that several reprocessing cycles need not, by themselves, impair impact strength or heat resistance [58], but post-consumer material of unknown history may behave differently. A data type property recording reprocessing cycles, and a rule that downgrades the quality grade when measured K-value retention falls below a grade limit, are therefore proposed as an extension (Section 12.5); they are not part of the 34 rules reported here.

### 5.5. Environmental and Carbon Indicator Representation

Environmental indicators are formalised using the following components: impact category (global warming potential in kg CO_2_e/kg recyclate), life cycle stage (collecting → sorting → cleaning → shredding → recycling), allocation technique (economic allocation, mass allocation, system expansion) and carbon factors database (Ecoinvent [74], DEFRA [110], literature). The carbon accounting process involves identifying direct emissions (combustion of process fuels), indirect emissions (electricity use), supply chain emissions (transportation, chemicals, packaging), and displaced emissions (displaced virgin production).

### 5.6. Cost, Value Recovery and Economic Variable Representation

The cost ontology includes capital expenditure (CAPEX: equipment purchase, installation, and commissioning, in € per tonne of annual capacity), operating expenditure (OPEX: labour, energy, consumables, maintenance, and quality testing), and transaction costs (collection fees, transport, gate fees, and compliance). Value recovery is formalised through secondary product prices (€/t, depending on quality grade), by-product valorisation (metals and paper fibre recovered from the waste stream), and gate fee revenue (€/t received for waste acceptance).

Virgin PVC is produced from ethylene, derived from petroleum, natural-gas liquids, or bio-based sources, and from chlorine produced by electrolysis of sodium chloride; both steps are energy-intensive, so virgin resin prices, and, with them, recyclate prices, follow feedstock and electricity markets. The framework therefore treats the virgin price and the recyclate price as scenario inputs to the economic ontology, rather than as constants: the recyclate price enters as a price per quality grade, and the virgin baseline enters the avoided-emission credit and the abatement-cost rule. Section 9.6 quantifies the consequence by showing, for each route, the probability that operating cost is recovered at a given recyclate price. When prices fall, routes with the highest operating costs lose viability first, which is why the rules compare routes on a like-for-like basis and do not treat any route as viable in isolation.

Table 5 presents representative cost structures.

### 5.7. Circularity and Net-Zero Decision Rules

Circularity rules encoded in the ontology include loop closure (if recyclate_quality ≥ secondary_specification then closed-loop feasible), downcycling assessment (if recyclate_quality < primary_specification then open-loop only) and residue management (if residue_rate > 0.10 then recovery technology review indicated). Net-zero rules include carbon abatement cost (if (cost_recycling − cost_virgin)/(emissions_virgin − emissions_recycling) ≤ carbon_price then economically efficient decarbonisation) and technology readiness (if TRL ≥ 7 and emissions_reduction ≥ 0.70 then near-term deployment priority).

## 6. Big Data Analytics Framework for Circularity Assessment

### 6.1. Process Data Analytics

Process data analytics applies statistical process control (SPC) to identify deviations from optimal operating conditions. Key metrics include temperature profiles (hot-end to cool-end delta, melt temperature stability with a target of ±5 °C), pressure stability (die pressure coefficient of variation with a target of less than 5%), current draw (motor load variation within ±10% of set point) and throughput (instantaneous versus average rate, downtime incidents). Anomaly detection algorithms flag conditions associated with quality degradation or excessive energy consumption. Statistical process control and anomaly detection are established data analytics methods [47,48].

### 6.2. Operational Performance Analytics

Operational analytics track productivity and efficiency metrics. Overall equipment effectiveness (OEE) is calculated as availability × performance × quality, with availability target above 85% (scheduled production time minus downtime), performance target above 90% (actual throughput divided by theoretical throughput) and quality target above 95% (good output divided by total output). Energy efficiency is measured as specific energy consumption in kWh/kg processed, with a baseline of 0.5–1.0 kWh/kg for mechanical recycling and opportunities for improvement through heat recovery and motor efficiency. Water efficiency is assessed through the closed-loop water recycling rate and fresh water consumption per tonne. Such indicators are typical of data-driven smart manufacturing [50,51].

### 6.3. Business and Cost Data Analytics

Business analysis considers costs and revenue in analysing profit. Contribution margin analysis is based on revenue minus the variable costs of a certain product grade. Cost analysis divides costs into fixed and variable costs in relation to cost centres and activities. Sensitivity analysis measures the effect of fluctuations in input costs (power, labour, materials). Scenario analysis performs what-if analysis for variations in feedstock composition and technology. Cost structures and market prices are taken from the techno-economic literature [38,39].

### 6.4. Environmental and Carbon Data Analytics

The metrics used in environmental analytics involve the computation and monitoring of sustainability metrics via carbon footprint calculation (activity data × emission factors, aggregating process step impacts), hotspot detection (those process steps having more than 20 per cent contribution to the total footprint will be analysed further), benchmarking (facility performance relative to industry averages, best-in-class, and regulatory requirements), and trend analysis (week-to-week and month-to-month carbon intensity trends). The calculation approach follows ISO 14067 [72].

Table 6 presents illustrative carbon intensities for PVC-u recycling routes.

### 6.5. Pattern, Trend and Anomaly Identification

Methods employed in analytics include the use of CUSUM and EWMA control charts for process drift detection to give early indication of quality degradation, k-means clustering for energy profiling to classify energy regimes in terms of energy efficiency, random forest regression for predicting the quality, support vector machines for classifying the feasibility of suggested recycling routes, and isolation forests for anomaly detection of unusual process states, all of which are well-recognised big data analytics methods [47,48].

**Figure 2:**
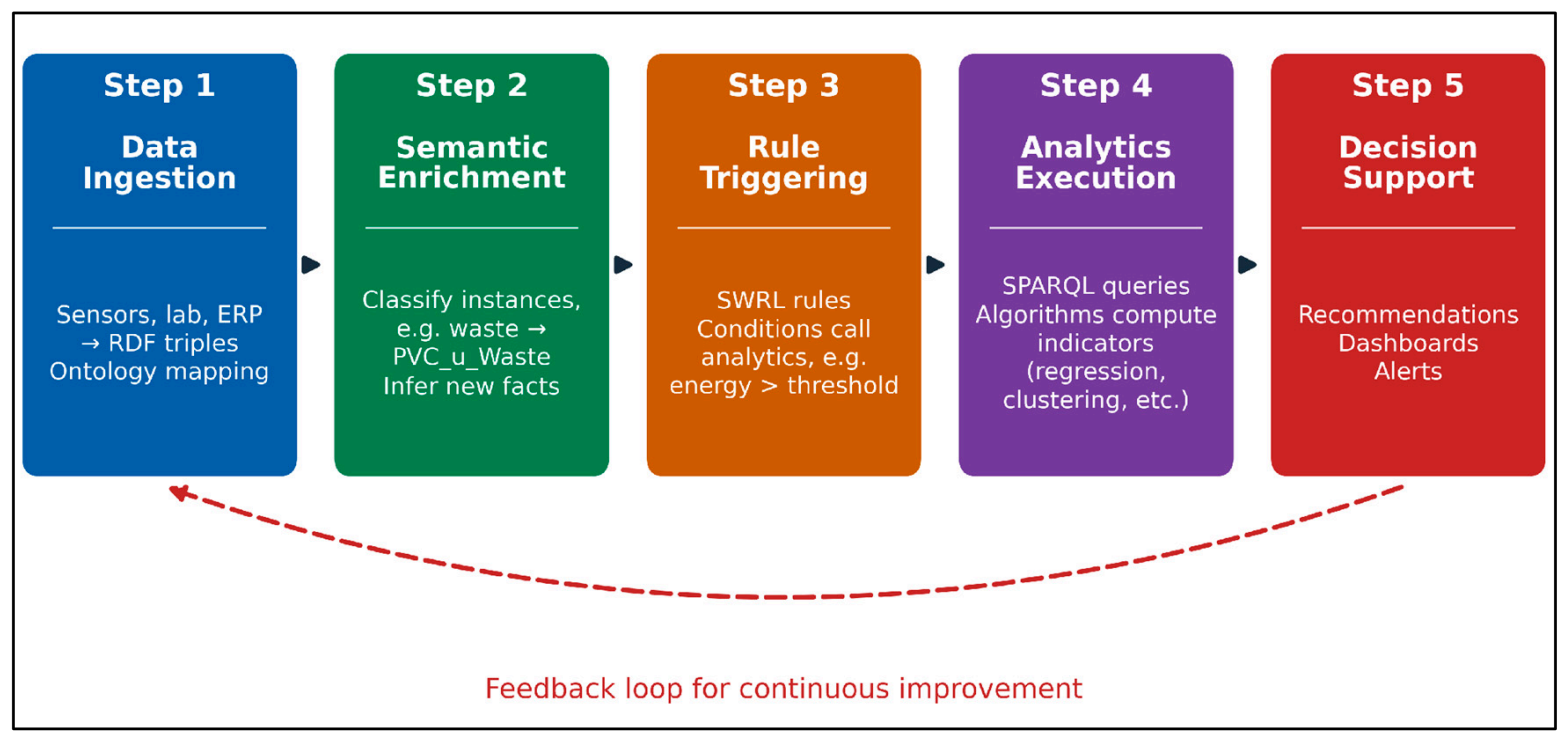
Ontology-linked analytics workflow from data ingestion to decision support with feedback loop to using outcomes to guide improvement.

Figure 2 illustrates the ontology-linked analytics workflow from data ingestion to decision support.

### 6.6. Ontology-Linked Analytics Workflow

The ontology-based analytics process consists of five stages. The first stage involves data ingestion: data from sensors, lab results, and the ERP system is mapped into RDF triples based on ontologies. The second stage is known as semantic enrichment: data instances are categorised into classes from the ontology (for example, a waste batch is categorised as PVC_u_Waste). The third stage includes rule triggering, where SWRL rules determine when it is necessary to conduct analytics (for example, when energy intensity is higher than a certain level). The fourth stage is about analytics processing: SPARQL queries are used to extract appropriate instances, and algorithms calculate indicators. The fifth stage is decision support. Results are instantiated back into the ontology, and human-readable recommendations are generated.

## 7. Micro-Level Circularity Decision Framework

### 7.1. Plant-Level Efficiency Indicators

Plant-level efficiency is assessed through material yield (output in kg divided by input in kg, target above 85%, data source production records), energy intensity (kWh per tonne processed, target below 800 kWh/t, data source utility meters), water intensity (m^3^ per tonne processed, target below 0.5 m^3^/t, data source flow meters), labour productivity (tonnes per person-hour, target above 0.5 t/person-hr, data source time tracking) and first-pass quality (percentage meeting specification without rework, target above 90%, data source quality testing).

### 7.2. Energy, Waste and Emissions Performance

Energy performance improvement rules encoded in the ontology include: if specific energy consumption exceeds 1.2 times the baseline and melt temperature exceeds 210 °C, then reduce the temperature setpoint (risk of degradation); if specific energy consumption exceeds 1.2 times the baseline and throughput is below 0.8 times nominal, then investigate feedstock feeding or screw design; and if specific energy consumption exceeds 1.2 times the baseline with no other cause, then schedule an energy audit. Waste metrics include residue rate (non-recyclable output mass divided by input mass; target below 10%), reject rate (failed quality inspections; target below 5%), and fines generation (particle loss below 1 mm; target below 3%).

### 7.3. Recyclate Quality and Process Yield

Table 7 presents quality grades and associated applications.

Improving yield procedures recommend that if the yield is less than 80% and the residue contains more than 5% PVC, the separation parameters should be changed; if the yield is less than 80% and rejections are greater than 10%, the quality specifications should be checked for their compatibility with the use; and if the yield is less than 80% and the throughput is normal, contaminations should be examined in the feedstock.

### 7.4. Cost, Productivity and Value Recovery

**Figure 3:**
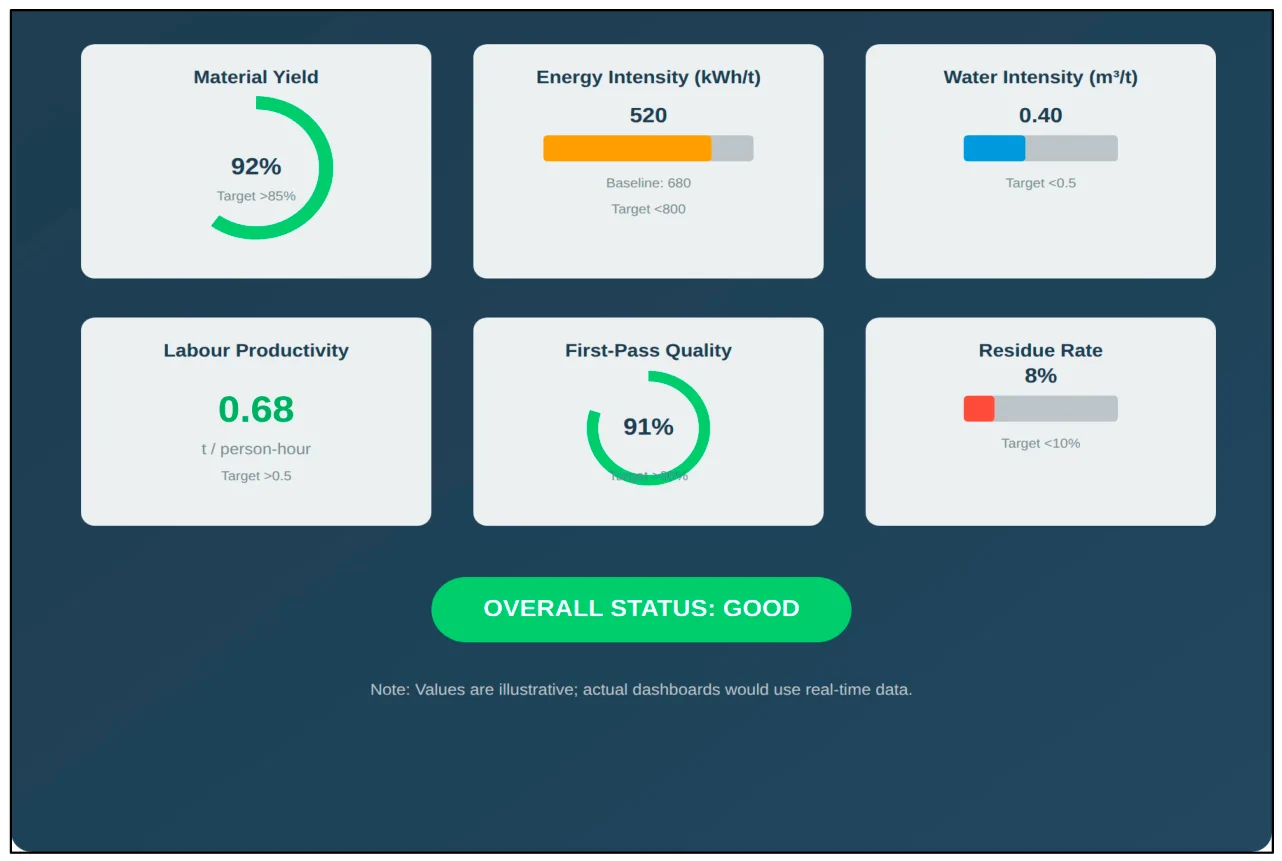
Conceptual dashboard of micro-sustainability indicators (plant-level KPIs).

Cost decision-making at the micro level involves marginal analysis. Good tonne cost is total OPEX/(production × quality rate). Value recovery efficiency is the sales value of secondary products divided by the total value of materials. Improvement priority is decided on a high-impact-first basis (in this case, the hierarchy of improvements would be energy improvement, yield improvement, labour improvement, since that is the order of impact in terms of costs in mechanical PVC-u recycling). Process optimisation rule of thumb: if the cost per tonne exceeds the achievable market price, reduce energy consumption and increase yield/quality (Figure 3).

### 7.5. Decision Logic for Operational Improvement

Continuous improvement is implemented through this model via real-time measurement (dashboard with key performance indicators against target values), warning conditions (SWRL rules that trigger warnings, for instance, if energy_intensity > threshold value), cause analysis (SPARQL queries to retrieve correlated variables), recommendation for action (ruleset-based recommendations, like “clean die head-pressure instability”) and outcome measurement (measurements of indicator changes post-action).

## 8. Macro-Level Circularity Decision Framework

### 8.1. Circularity and Resource-Retention Indicators

Macro-level circularity indicators comprise the circular material use rate (CMUR), which is calculated by dividing the amount of recycled input by the total material input, with an EU-wide value of 12.2% in 2024 [113] against an EU target of 22.4% by 2030 [114]; end-of-life recycling rate (EOL-RR), which is calculated by dividing the amount of recycled waste by the total waste produced with reference values from PlasticsEurope statistics [115] and a benchmark of 55%, which is the EU 2030 recycling target for plastic packaging [116] and is used here as an illustrative reference because no equivalent binding target exists for rigid PVC-u construction waste, where the sector commitment is expressed in tonnes recycled per year [11]; material circularity indicator (MCI), which is calculated following the Ellen MacArthur Foundation methodology [117] as MCI = 1 − LFI × F(X), where the linear flow index LFI = (V + W)/(2M + (WF − WC)/2) depends on the mass of virgin feedstock (V), of unrecoverable waste (W) and of the product (M), WF and WC are the waste generated in producing recycled feedstock and in recycling the product after use, and the utility factor is F(X) = 0.9/X; the range of 0.3–0.6 is used here as an illustrative reference band and is not an Ellen MacArthur Foundation target; and resource retention (RR), which is calculated as (Lin − Lout)/Lin × 100% targeting over 80% as an illustrative benchmark, where Lin denotes the amount that enters the system and Lout denotes the amount that leaves the system for disposal.

### 8.2. Industrial Value-Chain Implications

Macroscale decisions involve value chain design, which includes centralised recycling (facilities of 50,000 t/year or greater, low cost but large transport distance), decentralised pre-processing (sorting and density separating at a regional level, long-distance transport to further processing site) and vertical integration (relationships from recycler to compounder to manufacturer). The decision rules for value chain design are: if waste arisings of PVC-u exceed 100,000 t/year in a radius of 200 km, then assess the option of centralised mechanical recycling; if the average collection distance is greater than 500 km, then pre-process (density separate and agglomerate) before long-distance transport; and if the end-market demands 90% property retention, then use chemical recycling instead of mechanical.

### 8.3. Net-Zero Implementation Perspectives

The net-zero decision framework evaluates baseline emissions (current recycling system carbon footprint), abatement options (technology substitution, energy decarbonisation, efficiency gains), marginal abatement cost (€/t CO_2_e reduced) and technology maturity (R&D → pilot → demonstration → commercial). Table 8 illustrates abatement options for PVC-u recycling. The carbon price of €80/t used in the marginal abatement cost comparisons is close to the EU Emissions Trading System allowance price of about €83/t observed in late August 2026 [118]. Table 8 ranges are literature-informed and illustrative, and their interaction with carbon price uncertainty is examined in Section 9.6.

**Figure 4:**
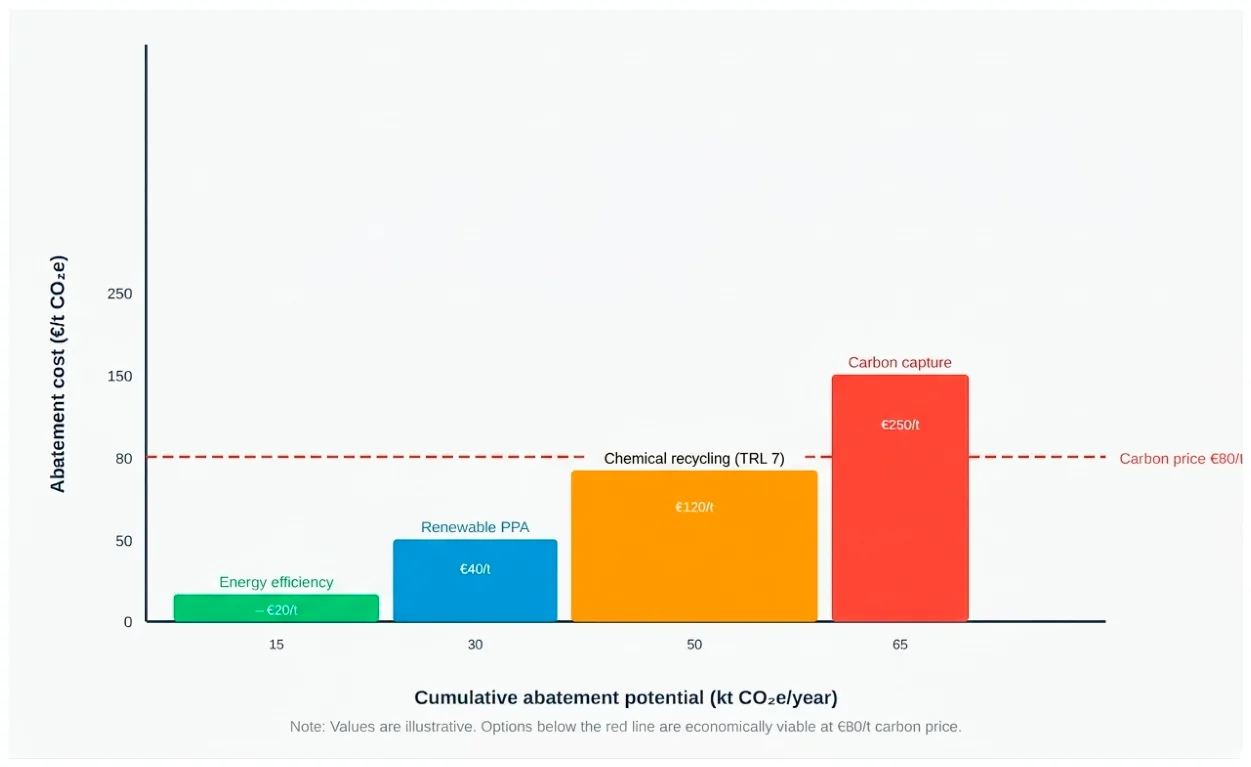
Marginal abatement cost curve for PVC-u recycling technologies. Options below the carbon price line (€80/t) are economically viable.

Figure 4 presents the marginal abatement cost curve for PVC-u recycling technologies.

### 8.4. Policy, Standardisation and Industrial Scalability

The policy alignment issues cover the EU Waste Framework Directive [121], which sets the targets for preparing for re-use and recycling, including at least 70% by weight for non-hazardous construction and demolition waste; the Construction Products Regulation [12], which introduces sustainability and digital product-information requirements for construction products; the REACH Regulation [22], whose restriction of lead in PVC [21] governs whether recovered rigid PVC may be placed on the market; and the Packaging and Packaging Waste Regulation (PPWR) [122], with mandatory recycled content levels of 10–35% by 2030 for plastic packaging. The PPWR applies to packaging and is therefore not directly applicable to rigid PVC-u construction products; it is included as a reference for recycled-content rules likely to influence recyclate demand, whereas the Waste Framework Directive, the Construction Products Regulation, and REACH are the instruments directly relevant to rigid PVC-u.

Standardisation deficiencies to be addressed include quality standards for recycled PVC-u material, rules for calculating the carbon footprint of recycled polymers, and guidelines for verifying circularity content claims.

### 8.5. Decision Logic for Wider Circular Economy Impact

Macro-level decision logic is governed by two principles. The first principle states that if the CMUR is below the national standard and the EOL-RR is below the objective, policy tools such as EPR and deposits/taxes should be considered. The second principle states that if the abatement cost is less than the carbon price and the technology’s TRL is greater than or equal to the deployment minimum, then invest.

## 9. Results and Framework Demonstration

### 9.1. Ontology Model Outputs

The Protégé ontology consists of 156 classes (6 top-level, 42 second-level, and 108 third-level), 84 object properties (transitive, symmetric, and inverse), 62 data properties (integer, float, string, boolean, dateTime), and 34 SWRL rules. Table 9 shows coverage of the ontology.

### 9.2. SWRL and SPARQL Reasoning Outputs

The results of applying example rules are as follows. Rule R01 (feasibility assessment) had input of PVC-u waste that contained 8% CaCO_3_ filler, 0.5% Pb stabiliser and 3% polyolefin contamination. The result suggested mechanical recycling after froth flotation for polyolefin removal and a lead-management step (routing to the permitted rigid construction applications with the required labelling, or stabiliser extraction), because 0.5% lead lies between the 0.1 wt% marking threshold and the 1.5 wt% derogation ceiling [21]. Rule R02 (quality prediction) had an input of a melt flow index of 4.2 g/10 min and a colour ΔE* value of 7.2. The result classified the product as Standard grade and proposed its use for drainage pipes. Rule R03 (carbon abatement prioritisation) used an input of existing carbon emissions of 500 kg CO_2_e/t and a carbon price of €80/t. The result prioritised energy efficiency (negative costs) first, then renewable energy purchase agreement (€40/t), and then chemical recycling (€120/t).

Chemical recycling (€120/t) is ranked third but exceeds the €80/t carbon price and would therefore not pass the economic-efficiency test of Section 5.7 at that price; it is retained as a strategic option to be revisited if the carbon price rises.

**Figure 5:**
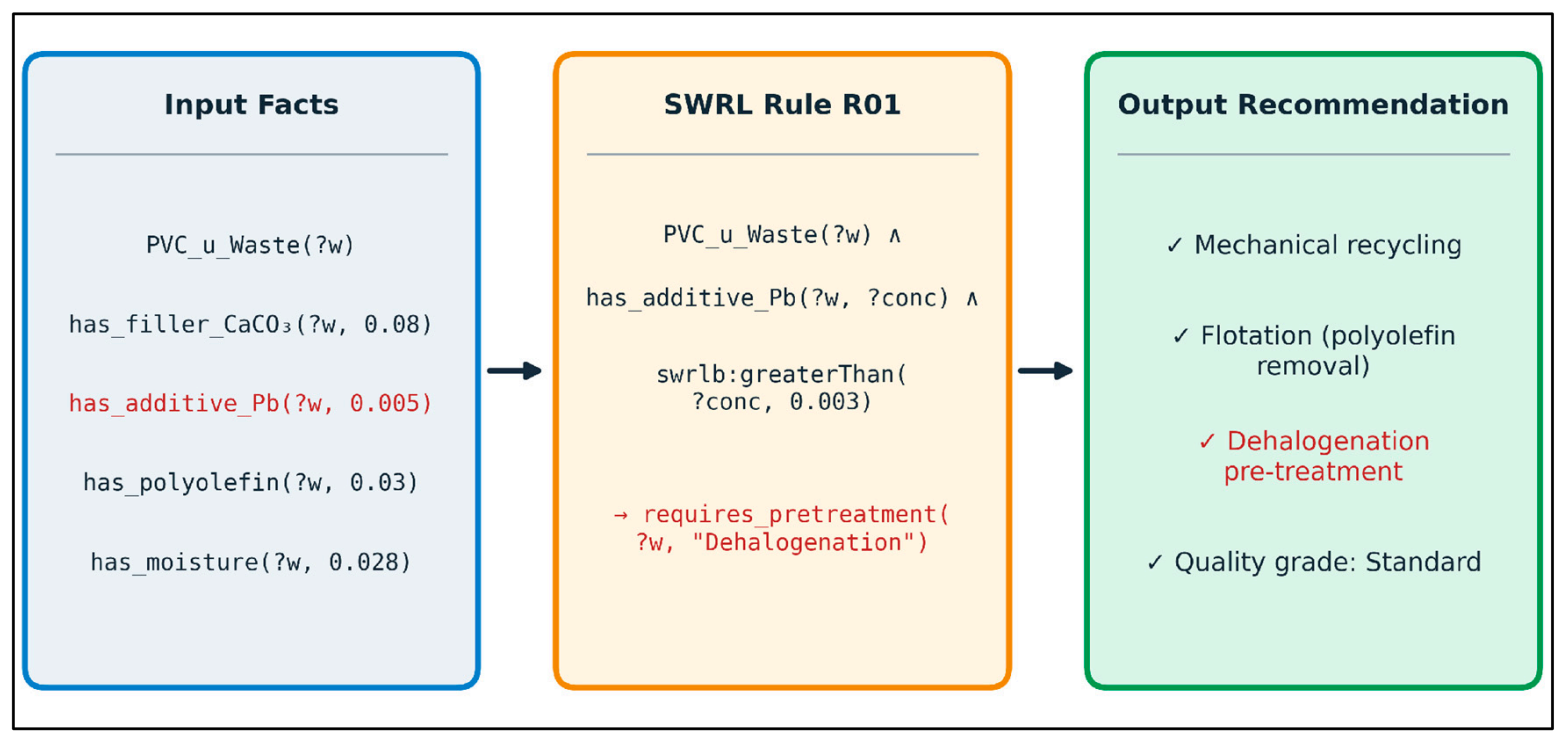
SWRL Rule Execution Example (Rule R01). Variables prefixed with a question mark (e.g., ?w, ?conc) are SWRL variables that are bound to individuals or data values at inference time; ?w denotes a PVC-u waste batch and ?conc the lead (Pb) additive concentration retrieved from the asserted facts. The wedge symbol (∧) denotes logical conjunction, meaning that all atoms in the rule antecedent must be simultaneously satisfied for the rule to fire. The arrow (→) separates the antecedent (body) from the consequent (head), which is asserted only when the antecedent holds. Check marks (✓) in the output panel indicate the process recommendations inferred and returned by the reasoning engine for the given input. Red text highlights the elements that trace the rule’s firing: the input fact has_additive_Pb(?w, 0.005), which exceeds the swrlb:greaterThan threshold of 0.003, the resulting assertion requires_pretreatment(?w, “Dehalogenation”), and the corresponding dehalogenation pre-treatment in the output recommendation (author’s own elaboration).

Figure 5 shows the SWRL rule execution example.

### 9.3. Sustainability and Economic Indicator Computation

Table 10 presents computed indicators for three illustrative recycling scenarios.

Scenario A is clean pipe offcuts with a known additive package and short transport. Scenario B is mixed construction waste with 670 km collection and 5% contamination. Scenario C is WEEE containing brominated flame retardants with a solvent purification step. All values in Table 10 are synthetic scenario values and not measurements. They align with the literature-informed ranges in Table 5 and Table 6, except that the carbon footprint of Scenario B (420 kg CO_2_e/t) slightly exceeds the process-emission range in Table 6 (200–400 kg CO_2_e/t); we interpret this as including emissions from the 670 km collection distance.

### 9.4. Micro-Level Decision-Support Results

For Scenario B, which involves mixed post-consumer waste, the framework has produced the following micro-level decision outputs. The conditions were that yield fell from 85% to 71% over three weeks, and energy intensity rose 22% above the target. The framework analysis revealed, via a SPARQL query, a relationship between the decrease in yield and polyolefin contamination, with the latter exceeding 4%. The SWRL rule was triggered such that when polyolefin_content > 0.02 (the 2% flotation trigger of Section 5.4) and yield < 0.80, then recommend_flotation_separation. It was also found that the moisture content was 2.8%, compared to the target of 1%. The recommendation was to install a froth flotation column for polyolefin separation and a pre-drying step. The expected effects are model-derived scenario estimates and not measured results. The yield improvement of +12%, interpreted here as 12 percentage points (71% to 83%), would recover about 86% of the 14-point loss. The energy reduction of 8% should be read as an upper-bound assumption: a simple thermodynamic check shows that evaporating the excess 1.8 wt% of moisture requires only about 13 kWh/t, roughly 2% of the scenario energy intensity, so the remainder of the assumed saving would have to come from effects that the scenario does not quantify. The economic consequence was a CAPEX of €450k and a payback period of 14 months, which implies an annual net benefit of about €386k, driven mainly by the improvement in yield value. If the realised benefits were 75%, 50% or 25% of the projected values, and benefits scale in proportion, the payback period would lengthen to about 19, 28 and 56 months, respectively. Confirming these estimates requires a pilot trial on a real line.

### 9.5. Macro-Level Decision-Support Results

Based on the baseline assessment, the current CMUR is 18% (below the EU goal of 24%), the EOL-RR is 42% (below the 55% benchmark), and the carbon footprint is 510 kg CO_2_e/t. The framework assessment developed an abatement curve based on 4 technologies, identified the marginal abatement cost of each of these technologies and developed an investment priority order consisting of the following three actions in this order: improvement of energy efficiency in existing lines, extended collection to collect 8000 t/year more and the implementation of a pilot solvent-based process for WEEE fractions, whose marginal abatement cost of €85/t in Scenario C marginally exceeds the €80/t carbon price, so that it is justified as a learning and option-value pilot and not as an economically efficient abatement measure at current prices. The projected 2030 scenario outcomes are CMUR 28%, EOL-RR 58%, emissions of 290 kg CO_2_e/t (a reduction of 43%), and an investment of €8.2 million over five years. These are target-consistent scenario outcomes and not forecasts: Section 9.6 shows that the CMUR and EOL-RR targets are met only if at least about 60% and 81%, respectively, of the projected improvement is realised, and that the projected pace of about 2 percentage points of CMUR per year is roughly 19 times the EU-wide trend of about 0.1 percentage points per year between 2010 and 2024 [113,114].

**Figure 6:**
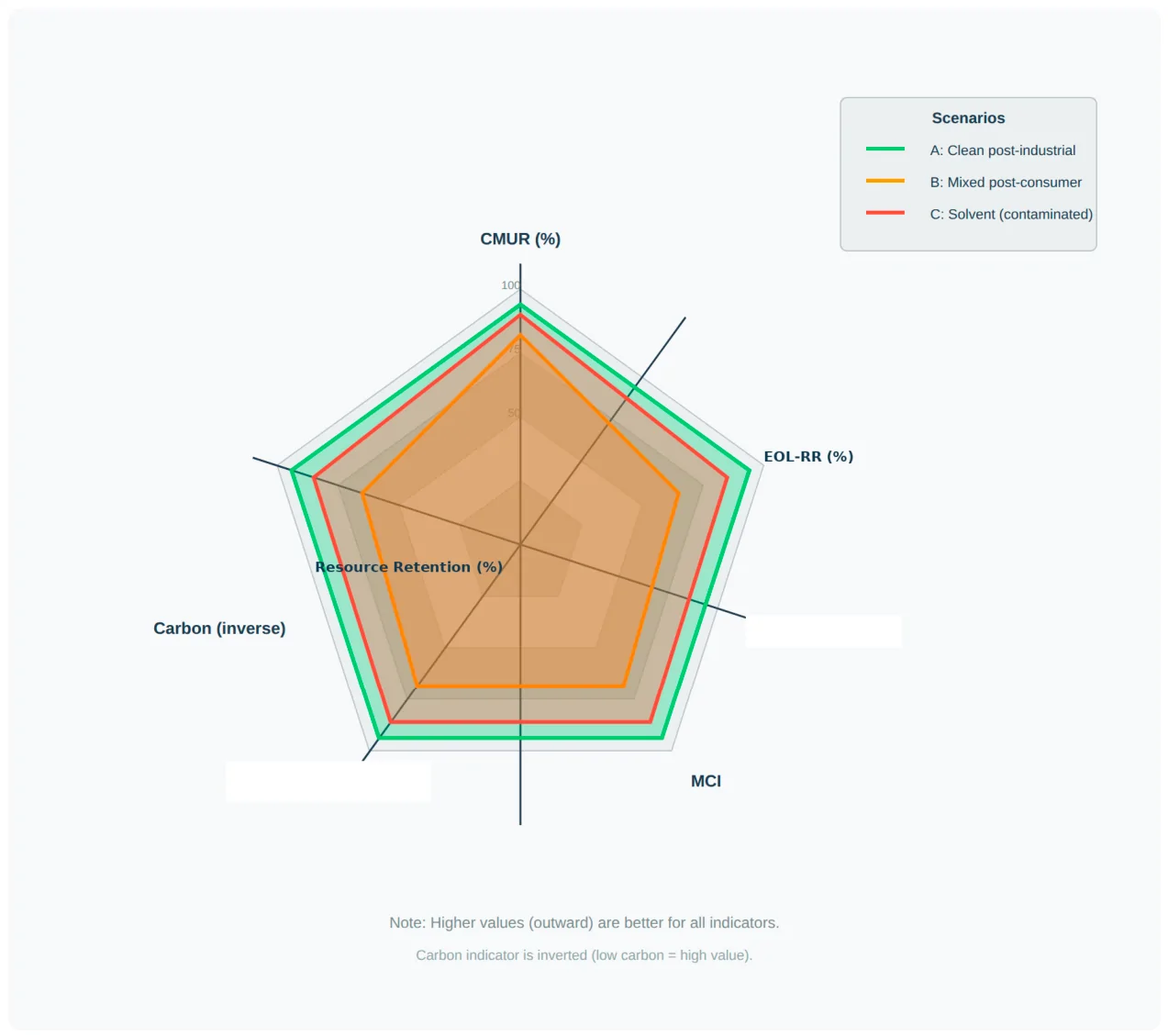
Radar chart comparing circular economy indicators across three recycling scenarios (A: clean post-industrial, B: mixed post-consumer, C: solvent-based) (author’s own elaboration).

Figure 6 presents a radar chart comparing circular economy indicators across the three scenarios.

### 9.6. Uncertainty, Sensitivity and Robustness Analysis

The scenario results above are point estimates on illustrative inputs. To show how uncertainty in those inputs propagates to the conclusions, and how robust the rule outputs are to measurement noise, four analyses were carried out with a Monte Carlo procedure (100,000 draws, fixed random seed, Python (version 3.15.0) and NumPy (version 2.5.0) [123,124]. Parameters for which the manuscript gives only a minimum and a maximum (Table 5, Table 6 and Table 8) were sampled from uniform distributions, the maximum-entropy choice for a bounded range, and were assumed independent. Assumptions that are not taken from the tables, namely the carbon-price distribution, the measurement-noise levels and the degree to which projected improvements are realised, are labelled as assumptions and are varied and not asserted. This is an uncertainty-propagation exercise on synthetic inputs; it is not a statistical validation against empirical data, which requires plant data (Section 11).

**Figure 7:**
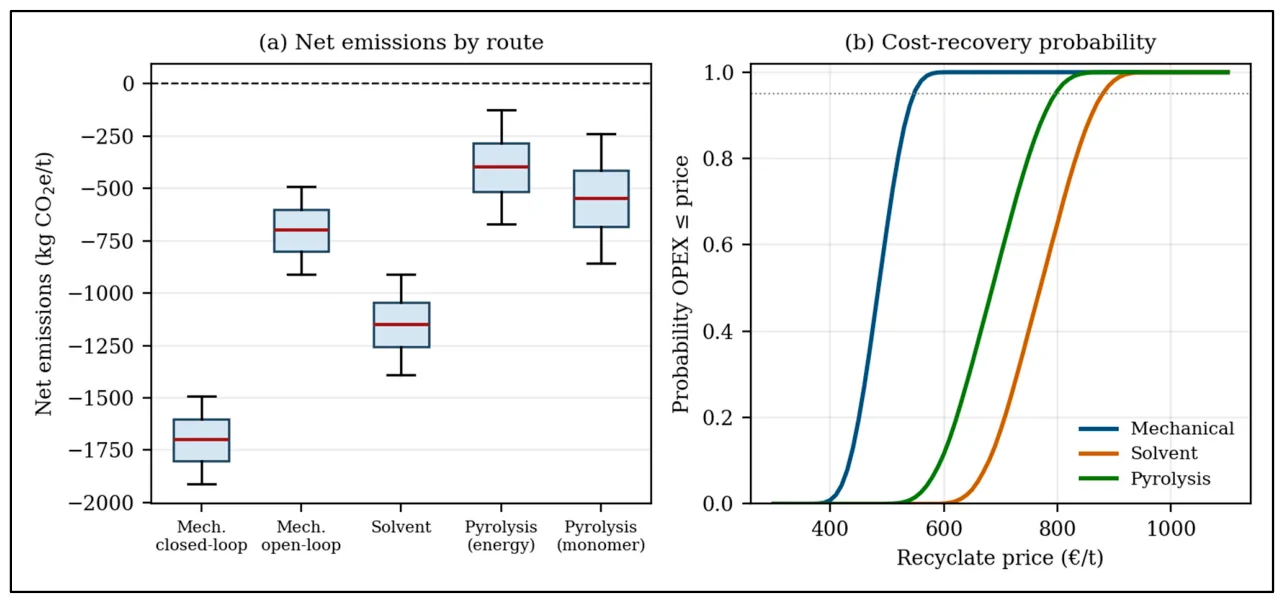
Uncertainty analysis of the illustrative scenario inputs (100,000 Monte Carlo draws, uniform distributions over the ranges of Table 5 and Table 6). (**a**) Net emissions by recycling route (box: interquartile range; line: median; whiskers: 5th to 95th percentile). (**b**) Probability that operating cost is recovered as a function of recyclate price (operating cost only).

Net emissions by route. Table 11 and Figure 7a show the distribution of net emissions (process minus avoided emissions) for each route. Every route remains a net emission saver across the full input ranges, but the savings differ by about a factor of four: the median saving is 1700 kg CO_2_e/t for closed-loop mechanical recycling, 1150 for solvent-based recycling, and 400 for pyrolysis with energy recovery. For pyrolysis with energy recovery, the upper bound is zero, so the route can only just break even on climate benefit under unfavourable inputs. The credit for avoided virgin production contributes more to the variance of net emissions than process emissions (rank correlations of 0.61 to 0.90 with the avoided-emission input), underscoring the importance of the substitution assumption in the system boundary of Section 4.7.

With regard to operating cost and cost recovery, because the OPEX total in Table 5 is the sum of the category extremes, it overstates the plausible range when categories vary independently: the 5th to 95th percentile ranges are €422–547/t for mechanical, €659–880/t for solvent-based and €574–796/t for pyrolysis routes (Table 12). Primary-process cost dominates the variance in every route (rank correlation 0.76 for mechanical and 0.86 for the chemical routes), so the economic case for any route rests mainly on the reliability of process-cost estimates. Figure 7b shows the probability that operating cost is recovered at a given recyclate price (operating cost only; capital cost, gate fees and by-product revenue are excluded). At €550/t mechanical recycling recovers its operating cost in 96% of draws, whereas the solvent-based and pyrolysis routes do so in 0% and 2%; at €700/t the figures are 100%, 17% and 57%, and at €800/t they are 100%, 65% and 96%. Even mechanical recycling recovers its operating cost in only 64% of draws at €500/t and 20% at €450/t. If raw-material prices fall and pull recyclate prices down, the chemical routes therefore lose economic viability first, and none of the routes remains secure without gate-fee income or carbon-price support once recyclate prices approach €500/t.

Abatement cost and carbon price. Sampling the Table 8 cost ranges against an assumed triangular carbon-price distribution of €60–120/t with a mode of €82/t (anchored on the observed allowance price of about €83/t in August 2026 [118]; the upper bound is a scenario value and not a forecast), energy efficiency and renewable electricity pass the abatement-cost test in all draws, chemical recycling in 13% and carbon capture on pyrolysis in none. For Scenario C, whose marginal abatement cost is €85/t, the carbon price exceeds that level in about 54% of draws (and €80/t in 70%), so the solvent-based pilot recommended in Section 9.5 is economically efficient in only about half of the assumed carbon-price outcomes and is better described as an option-value pilot.

Projected 2030 outcomes. The 2030 values in Section 9.5 are target-consistent scenario outcomes and not forecasts. Table 13 expresses them through a realisation fraction φ, the share of the projected improvement from the baseline that is actually achieved. The CMUR target of 24% is met if φ ≥ 0.60 and the EOL-RR target of 55% if φ ≥ 0.81, so the end-of-life recycling target is the more demanding. Sampling φ from a triangular distribution with mode 1 and upper limit 1.1 (an assumption), the probability of meeting the CMUR target is 84%, 97% and 100% for lower limits of φ of 0.3, 0.5 and 0.7, and the probability of meeting the EOL-RR target is 53%, 67% and 89%. A plausibility check against observed data also counsels caution: the projected gain of 10 percentage points of CMUR over the five-year investment horizon implies about 2 percentage points per year, whereas the EU-wide circular material use rate rose from 10.7% in 2010 to 12.2% in 2024, about 0.1 percentage points per year [113,114]. A facility- or region-level system is not directly comparable with an EU-wide average, but the comparison indicates that the projection assumes a step change in collection, sorting and demand for recyclate.

To illustrate the threshold provenance and sensitivity, Table 14 documents the origin of the principal decision thresholds and whether the scenario decisions would change if a threshold were shifted. Regulatory thresholds (lead, carbon price) have an external basis, whereas the contamination, yield and energy thresholds are illustrative practitioner heuristics that must be calibrated with plant data. Decisions with wide margins, such as lead at 0.5 wt% against the 0.1–1.5 wt% band, are insensitive to moderate threshold changes, whereas two are fragile: the total contamination of Scenario B (5%) lies exactly on the Low_Grade boundary (>5%), and its yield (78%) lies only two percentage points below the 80% yield trigger.

Real plant data are noisy, whereas the synthetic inputs are exact. To test how far the rule outputs depend on that exactness, each threshold input was perturbed with multiplicative Gaussian noise with a coefficient of variation of 5%, 10%, 20% and 30% (an assumed range from low to severe scatter), and the share of draws that reproduced the noise-free decision was recorded (Table 15). Rules with wide margins are robust: the flotation decisions for R01 and Scenario B and the lead-band decision are reproduced in at least 87%, 95% and 100% of draws, respectively, even at 30% noise, and the Standard grade in R02 (ΔE* = 7.2) holds in at least 75% of draws. Rules close to a threshold are not: the Low_Grade decision for Scenario B is a coin toss (about 50% of draws) whatever the noise level, and the yield trigger for Scenario B (78% against 80%) is reproduced in only 53% to 70% of draws. Implementations should therefore evaluate borderline cases with a tolerance band, flagging results that lie within one measurement standard deviation of a threshold for review instead of switching decisions abruptly.

### 9.7. Interpretation of Circular Smart Manufacturing Outcomes

The demonstration of the framework supports four findings, within the limits of synthetic data and of the uncertainty analysis in Section 9.6. To begin with, ontological expressiveness is sufficient to represent decision-relevant relationships concerning materials, processes, quality, sustainability, and economics. Secondly, SWRL rules are useful for capturing decision-making logic for quality classification, process selection, and investment prioritisation. Thirdly, micro-macro integration is achieved through indicator traceability, in which plant-level energy intensity contributes to regional circularity indicators. Lastly, economic integration is implemented using abatement-cost calculations that link environmental outcomes to investment decisions. Section 9.6 adds a fifth, cautionary finding: rules whose inputs lie close to a threshold, such as the contamination limit of Scenario B, are unstable under measurement noise, so real deployments need borderline bands as well as hard thresholds.

## 10. Discussion

### 10.1. Contribution to Rigid PVC-u Recycling Research

This study makes three contributions to PVC-u recycling research. First, it combines separate areas of knowledge in one framework, addressing the fragmentation noted in the literature [1,2,3,23,25]. The ontology links material properties, processing parameters, quality results and sustainability indices, which were previously treated separately. Second, the framework addresses the legacy additive problem [8,9,10] through rules that determine how contaminants affect recyclability, and it distinguishes closed-loop from open-loop outcomes according to additive type. Third, the framework connects the mechanical and chemical recycling literature [6,17,25,62] by comparing recycling routes within a single decision scheme. It covers both established mechanical processes and emerging chemical technologies at different technology readiness levels.

### 10.2. Contribution to Ontology-Based Sustainability Decision Support

The framework advances ontology-based sustainability decision support in three ways. First, it extends materials ontologies with sustainability and economic concepts relevant to circular economy decisions [42,43]; second, it embeds rule-based decision logic in SWRL [82] to implement sustainability assessment criteria derived from various approaches such as LCA [30,34,71], circular economy models [32,79] and industrial practices; and third, its analytics show how ontologies can help create, and not only describe, sustainability knowledge. The rules that link data anomalies to decision recommendations are a distinctive contribution. Compared with earlier manufacturing ontologies [44,84], this approach focuses on end-of-life rather than manufacturing contexts, an under-researched area in semantic knowledge representation.

### 10.3. Contribution to Smart Manufacturing Economics

The economic component addresses a known weakness of environmental decision models. It combines marginal abatement cost from climate economics, technology readiness level assessment from innovation management, and payback and NPV calculation from capital budgeting. This responds to the criticism that environmental assessments give too little guidance for sustainable investment decisions [38,78]. The economics layer extends Industry 4.0 theory [50,91,92] by specifying inputs, analysis methods and decision outputs for recycling operations, which differ from primary manufacturing in feedstock variability, quality uncertainty and multi-stakeholder value chains.

The uncertainty analysis in Section 9.6 allows the economic results to be read as decision-relevant and not merely descriptive. Under the ranges of Table 5, mechanical recycling has a median operating cost of about €485/t (5th to 95th percentile €422–547/t), against about €770/t for solvent-based and €686/t for pyrolysis routes. A recyclate price of €550/t therefore recovers operating cost in 96% of draws for mechanical recycling but almost never for the two chemical routes, which need prices of roughly €700–800/t or more. Because primary-process cost dominates the variance in every route (rank correlation of 0.76 to 0.86 with total cost), the economic case for any route rests mainly on the reliability of process-cost estimates and not on collection, sorting or quality-control costs. The chemical routes are therefore more exposed to falling virgin-resin prices than mechanical recycling. Their justification rests on quality, on feedstock that mechanical routes cannot process, and on carbon-price developments, not on cost parity, a pattern consistent with recent techno-economic assessments of chemical recycling of PVC-containing plastics [65]. At the carbon price used here, energy efficiency and renewable electricity pass the abatement-cost test in every draw. Chemical recycling passes it in only about 13% of draws under the assumed price range, and the Scenario C solvent pilot in roughly half. The payback result is similarly sensitive: the 14-month payback of Scenario B lengthens to about 28 months if only half of the projected benefit is realised. These figures follow from illustrative ranges, should be replaced by plant and market data in applications, and are not forecasts of profitability.

### 10.4. Industrial Application and Implementation Relevance

The framework is designed to be practical to implement. Its data requirements match the usual capabilities of recycling plants (sensors, laboratory analysis and ERP software), its modular architecture allows facility-specific extensions, its rule sets are transparent and can be updated as knowledge changes, and its analytics can be built with open-source software (Python or R) and SPARQL endpoints. Implementation paths differ by facility type. For a single-line mechanical recycler, the preferred initial application is quality prediction (Section 7.3), with prioritised rules R01 and R02 (feasibility, quality). For a multiprocess plant, the preferred initial application would be a technology comparison using prioritised rules R03 through R07 (route selection). For a waste management company, the preferred initial application would be macro-level circularity monitoring, with prioritised rules R15 through R20 (policy alignment). Before operational use, the rules and thresholds should be validated against plant data as set out in Section 11.

### 10.5. Implications for Circular Economy and Net-Zero Strategy

The framework supports the transition to a circular economy in three ways. It quantifies trade-offs between closed-loop recycling (high-value recovery, high processing effort) and open-loop recycling (lower specifications, lower processing effort). It converts indicators into KPIs, for example by translating a CMUR target into the percentage recycled at plant level. It also expresses regulatory obligations as rules that can be monitored on dashboards. The abatement cost curve supports investment prioritisation for a net-zero strategy by deferring costly options until market mechanisms such as carbon pricing mature.

## 11. Limitations and Validity Threats

Data validity. All quantitative results are derived from synthetic, literature-informed inputs (Section 4.2); no measurement from an operating plant has been used. The yield and energy effects, the payback period and the 2030 projections of Section 9 are therefore model-derived scenario estimates that have not been confirmed empirically, and the assumed 8% energy saving in particular exceeds what the moisture-related thermal load alone would explain. Validation with real plant data is the main outstanding task, and until it is done the results should be read as demonstrations of how the framework processes information.

Statistical treatment. The Monte Carlo analysis propagates parameter ranges that are themselves illustrative. It assumes uniform, independent inputs and assumed carbon-price, noise and realisation distributions, so it quantifies sensitivity to those assumptions and not the probability of real-world outcomes; correlations between inputs, for example between energy prices and process cost, are not modelled.

Model and rule validity. The 34 SWRL rules encode priority decision logic and do not cover all recycling options. Several thresholds are illustrative heuristics (Table 14), some scenario inputs lie close to thresholds and are unstable under noise (Table 15), and the rules have not been compared with the decisions of plant experts. Quality degradation is represented through quality grades and not through the reprocessing history of the material, which the ontology does not yet record. Big data analytics are exercised here on small synthetic datasets: the term refers to the intended operating regime of high-frequency historian, laboratory and enterprise data and not to the scale of the demonstration, and the practicability of machine learning on real PVC extrusion data has been shown elsewhere [93].

Scope. The framework is designed for rigid PVC-u. Flexible (plasticised) PVC and mixed rigid-flexible feedstocks are not modelled (Section 1.1). The recovery of lead from residue sludges and landfilled deposits is not represented; lead-bearing residues are treated only as a residue stream. Social sustainability indicators are excluded, and the regulatory basis is European, so adaptation to other regions is needed. The cost analysis of Section 9.6 covers operating cost only, without capital cost, gate fees or by-product revenue, and the system boundary excludes the use phase and product conversion (Section 4.7).

Technology and epistemology. Scalability to ontologies of more than 1000 classes and real-time reasoning on high-velocity data streams have not been tested, and integration with proprietary ERP and MES systems requires adapters. The framework encodes current knowledge; rapid advances in chemical recycling and additive removal may outpace ontology updates, and contested definitions, such as the operationalisation of “circularity”, are embedded in particular framework choices.

Planned validation. A validation protocol is proposed for the industrial case studies described in Section 12.5: (i) collect, for example, twelve months of historian, laboratory and enterprise data from two to three recyclers; (ii) compare rule outputs with the decisions of plant experts on a blind set of cases and report agreement and misclassification rates; (iii) compare predicted quality grades and indicators with measured values using error metrics such as the mean absolute error and Cohen’s kappa for grade classification; (iv) recalibrate the thresholds of Table 14 from the data; and (v) fix acceptance criteria before testing.

## 12. Conclusions and Recommendations

### 12.1. Summary of Key Findings

This paper developed an ontology- and data-driven framework for circularity assessment of waste-derived rigid PVC-u and demonstrated it on three illustrative, literature-informed scenarios (clean post-industrial, mixed post-consumer and contaminated WEEE streams). The main outcomes are as follows. (1) Representation: the ontology of 156 classes and 84 properties captures the material, process, quality, sustainability, economic and decision-context relationships needed to support 34 SWRL decision rules. (2) Micro-macro integration: plant-level indicators (yield, energy intensity, quality grade) were aggregated into macro-level indicators (CMUR, EOL-RR, abatement cost) through predefined aggregation functions and traceability relationships. (3) Decision support: the SWRL rules for feasibility assessment, quality classification and investment priority produced consistent and traceable recommendations in all three scenarios. (4) Economics: marginal abatement cost calculations within the same framework ranked decarbonisation options by cost efficiency.

The uncertainty analysis of Section 9.6 refines and qualifies these findings. First, all five recycling routes remain net emission savers across the illustrative ranges, with median savings of about 1700 kg CO_2_e/t for closed-loop mechanical recycling and 400 kg CO_2_e/t for pyrolysis with energy recovery. Second, mechanical recycling recovers its operating cost at a recyclate price of €550/t in 96% of draws, whereas the solvent-based and pyrolysis routes require prices of roughly €700–800/t or more and are therefore the most exposed to falling raw-material prices. Third, the projected 2030 CMUR and EOL-RR targets are attained only if about 60% and 81%, respectively, of the projected improvement is realised, and the implied pace of CMUR improvement is far above the EU-wide trend. Fourth, rules whose inputs lie near a threshold are unstable under measurement noise. These are properties of illustrative inputs and not empirical findings; they indicate where empirical validation matters most.

### 12.2. Theoretical Contribution

The main theoretical contributions include the application of knowledge representation theory to circularity assessment in circular manufacturing in order to show that ontologies can represent not only the static knowledge of a domain but also decision-making processes and trade-offs; the integration model, which bridges micro and macro sustainability levels via traceability formalisation, thus bridging an acknowledged gap within methods for assessing circular economies; and the implementation of circular economy concepts as computable rules and indicators, moving beyond conceptual definitions to implementable decision support.

### 12.3. Methodological Contribution

Contributions to methodology consist of a methodology for the development of ontologies for recycling systems that includes formulating competency questions, defining classes, properties, and rules; a methodology for integrating ontology with analytics that consists of the ingest, semantic enrichment, rule activation, analytics execution, and result instantiation in a recycling workflow; and an indicator selection and operationalisation procedure that connects the sustainability assessment literature with industrial data.

### 12.4. Industrial and Policy Contribution

The framework can serve industry by providing structured knowledge creation and documentation for training and processes, consistent decision support with reduced dependence on unstructured expert opinion, and transparency in sustainability assessments for reporting to their customers. For policymakers, the framework is useful for simulating the impacts of policy instruments (for instance, the impact of a carbon price on abatement investment decisions), benchmarking facility operations against circular economy goals, and identifying gaps for R&D investment.

Policy recommendations. The results support the following recommendations, which should be read within the limits of synthetic data. (1) Set recycled-content and recycling targets for construction PVC directly: the targets used here are borrowed from plastic packaging and economy-wide indicators that do not reflect the long service lives and legacy additives of rigid PVC-u, and the Construction Products Regulation [12] is the natural vehicle. (2) Resolve the legacy-lead question through traceability: labelling and batch-level documentation of lead content under Regulation (EU) 2023/923 [21], together with digital product passports [90], would let recyclers route feedstock by measured lead content (Section 4.5), and the derogation that ends on 28 May 2033 should be reviewed in the light of lead-removal technology and of evidence that the restriction lowers recycling rates [20]. (3) Strengthen the carbon-price signal or provide targeted support for chemical and solvent-based routes: at a carbon price near €80/t, only energy efficiency and renewable electricity are economically efficient, and chemical recycling passes the abatement-cost test in a minority of the outcomes examined (Section 9.6). (4) Stabilise recyclate demand, for example through recycled-content requirements and green public procurement, because the routes with the highest operating cost are the most exposed to falling virgin-resin prices. (5) Support selective demolition, collection and sorting infrastructure, since the projected CMUR and EOL-RR gains depend on collecting substantially more material. (6) Fund open, comparable industrial validation, including shared data standards and ontology interoperability [88], so that decision-support tools can be tested against real plant data.

### 12.5. Recommendations for Future Research

Research objectives in the near term (1 to 2 years) will involve industrial case studies (validate the framework using two to three recyclers to test rules, improve indicators and evaluate performance), flexible PVC (extend the framework to plasticised PVC recycling including DEHP removal and plasticiser migration issues), and social sustainability criteria (extend indicators to address occupational health, community impact, and labour issues). Objectives in the mid-term (2 to 4 years) will involve updating the ontology dynamically (implement processes to update the ontology using emerging technologies like enzymatic dechlorination and advanced sorting systems), cross-polymer application (extend the ontology to include polyolefins, PET, and mixed streams), and open-source release (publish ontology OWL, rule sets, and analytics code). Long-term research objectives (4 to 6 years) will involve distributed reasoning systems (implementing ontology federation among supply chain members), machine learning (learning rule weights and indicator thresholds from operational data), and digital twin integration (implementing ontology synchronisation with facility digital twins in real time). Additional near-term extensions identified in this study are: a datatype property for reprocessing cycles with a downgrading rule for cumulative degradation (Section 5.4); a residue class for lead-bearing sludges and landfilled deposits with recovery, stabilisation and disposal options; explicit treatment of mixed rigid-flexible feedstock; feedstock-price scenarios and correlated inputs in the uncertainty analysis; tolerance bands for threshold rules; and a stakeholder study with recyclers to assess adoption.

### 12.6. Final Conclusions

The study set out to develop a decision-support framework that links plant-level operations to system-level circularity for waste-derived rigid PVC-u. It shows that a Protégé ontology coupled with rule-based reasoning and analytics can represent the relevant knowledge and evaluate it consistently in three illustrative scenarios. Under Monte Carlo uncertainty analysis, all five recycling routes remain net emission savers, mechanical recycling recovers its operating cost in 96% of draws at a recyclate price of €550/t, chemical routes do not, and the 2030 CMUR and EOL-RR targets are met only if about 60% and 81%, respectively, of the projected improvement is realised. Rules whose inputs lie close to a threshold are unstable under measurement noise, so borderline bands are needed alongside hard thresholds. Because all inputs are synthetic and literature-informed, these results demonstrate how the framework works and where validation matters most; they are not measured plant performance. The necessary next step is the validation protocol of Section 11, applied with two to three recyclers, followed by the extensions listed in Section 12.5.

## Figures and Tables

**Table 1:** Framework components and theoretical foundations.

Framework Component	Theoretical Foundation	Key References
Ontology knowledge base	Knowledge representation theory	[40,41]
Semantic reasoning (SWRL/SPARQL)	Rule-based inference	[82,83]
Big data analytics	Data science and machine learning	[47,48]
Micro-sustainability indicators	Manufacturing sustainability	[30,31]
Macro-sustainability indicators	Circular economy assessment	[32,79]
Decision integration	Multi-criteria decision analysis	[98]

**Table 2:** Primary ontology classes.

Top-Level Class	Subclasses
PVC_u_Material	PVC_u_Grade, Filler, Additive, Contaminant, Recyclate
Recycling_Process	Mechanical_Recycling, Chemical_Recycling, Solvent_Recycling
Quality_Attribute	Mechanical_Property, Thermal_Property, Rheological_Property, Optical_Property
Sustainability_Indicator	Environmental_Impact, Circularity_Metric, Social_Indicator
Economic_Variable	Cost, Revenue, Investment, Value_Recovery
Decision_Context	Micro_Decision, Macro_Decision, Strategic_Decision

Source: Author’s own elaboration based on ontology development following the methodology of Noy and McGuinness [101], with class structures informed by the literature on sustainable manufacturing ontologies [44,84] and materials ontologies [42,43].

**Table 3:** Indicator framework.

Indicator Type	Micro-Level Indicators	Macro-Level Indicators
Environmental	Energy intensity (MJ/kg), water consumption (L/kg), waste generation (kg/kg)	Cumulative energy demand (MJ), water depletion (m^3^), waste diversion rate (%)
Carbon	Process emissions (kg CO_2_e/kg), transport emissions (kg CO_2_e/kg)	Life cycle emissions (kg CO_2_e/kg recyclate), avoided emissions (kg CO_2_e)
Cost	Operating cost (€/kg), maintenance cost (€/kg), quality cost (€/kg)	Levelised cost of recycling (€/t), net present value (€), payback period (years)
Circularity	Recycled content (%), material yield (%), residue rate (%)	Circular material use rate (%), end-of-life recycling rate (%), material circularity indicator

**Table 4:** PVC-u grades and typical properties.

Grade	K-Value	Mw (kDa)	Mn (kDa)	Typical Application
K60	60–62	70–85	35–42	Pipe fittings, profiles
K65	65–67	90–110	42–52	Pressure pipes, window profiles
K67	67–70	110–130	50–62	High-impact applications

Source: Values are representative of typical PVC-u resin specifications. K-value ranges and molecular weight data are consistent with industry practice for suspension PVC resins; see, for example, the discussion of UPVC grades in the Gulf Standardisation Organisation [105] and the PVC resin specification literature. For determination of K-value, reference is made to ISO 1628-2 [106] and EN 922 [107] (Reference [107] is cited for historical provenance: EN 922:1994 established the viscosity-number procedure for calculating the K-value of PVC-U resins, the metric on which the K60–K67 grade designations in Table 4 are based. The standard has since been withdrawn and superseded by EN ISO 13229 [108], which is the current reference for new measurements; EN 922 is retained here only to document the origin of the K-value classification system).

**Table 5:** Representative cost structures for recycling routes (€/t).

Cost Category	Mechanical Recycling (€/t)	Solvent Recycling (€/t)	Pyrolysis (€/t)
Collection and transport	80–120	80–120	80–120
Sorting and pre-treatment	50–100	50–100	50–100
Primary process	150–250	300–500	200–400
Post-processing	50–100	100–200	100–200
Quality control	20–50	30–60	40–80
Total OPEX	350–620	560–980	470–900

Note: Costs are illustrative ranges based on typical techno-economic assessments in the literature [38,39]. The total OPEX row is the sum of the category minima and maxima (arithmetic bounds); Section 9.6 reports the probabilistic range obtained when the categories vary independently.

**Table 6:** Illustrative carbon intensities for PVC-u recycling routes (kg CO_2_e/t).

Recycling Route	Process Emissions (kg CO_2_e/t)	Avoided Virgin Emissions (kg CO_2_e/t)	Net Emissions (kg CO_2_e/t)
Mechanical (closed-loop)	200–400	1800–2200	−2000 to −1400
Mechanical (open-loop to lower grade)	200–400	800–1200	−1000 to −400
Solvent-based	500–800	1600–2000	−1500 to −800
Pyrolysis (with energy recovery)	800–1200	1200–1600	−800 to 0
Pyrolysis (to monomers)	1000–1500	1600–2000	−1000 to −100

Notes: Virgin PVC-u production baseline is 1800–2200 kg CO_2_e/t. Net negative indicates emissions savings compared to virgin. Net emissions are calculated using interval arithmetic (lowest process value minus highest avoided value, and highest process value minus lowest avoided value); probabilistic ranges are reported in Section 9.6. Source: Carbon intensity values are illustrative and informed by life cycle assessment studies of PVC recycling [65]. Virgin PVC production emissions typically range from 1.8 to 2.2 kg CO_2_e per kg of resin [23,39,111]. Mechanical recycling process emissions are derived from typical energy consumption and material loss rates. For comparative LCA data on PVC recycling routes, see the VinylPlus sustainability reports and the broader LCA literature on plastic recycling [34,35,75].

**Table 7:** Quality grades and recommended applications.

Quality Grade	K-Value Retention	Colour (ΔE*)	Mechanical Retention	Recommended Application
Premium	>95%	<5	>90%	Pipe core layer, non-pressure pipe
Standard	>85–95%	>5–10	>80–90%	Profile internals, drainage pipe
Utility	>70–85%	>10–15	>65–80%	Construction backing, filler
Reject	<70%	>15	<65%	Energy recovery

Note: Ranges are inclusive of the lower bound and exclusive of the upper bound (e.g., “Standard” K-value retention is ≥85% and <95%). Source: Quality grade definitions and recommended applications are informed by industry practice for PVC recyclate classification and the emerging European standard EN 18064-5 [109], which provides characteristics and typical values for PVC recyclates intended for defined application groups (product families). The threshold values for K-value retention, colour and mechanical properties are illustrative of typical quality assessment criteria used in PVC recycling operations [29,60,61]. In the colour column, ΔE represents the difference between two colours, usually a reference colour and a measured sample. The asterisk (*) is part of the conventional notation associated with CIELAB colour space, where colour coordinates are denoted by $L^{*}$, $a^{*}$, and $b^{*}$. ΔE* therefore, generally denotes the magnitude of the colour difference calculated from differences in these coordinates [112].

**Table 8:** Abatement options for PVC-u recycling.

Abatement Option	Emission Reduction Potential	Cost (€/t CO_2_e)	Technology Readiness	Priority
Energy efficiency (heat recovery)	15–25%	−10 to −30 (saving)	Commercial (TRL 9)	High
Renewable electricity PPA	40–60%	30–50	Commercial (TRL 9)	High
Chemical recycling (TRL 7–8 deployment)	50–70%	80–150	Demonstration (TRL 7)	Medium
Carbon capture on pyrolysis	70–90%	150–300	Pilot (TRL 6)	Low (2030+)

Source: Abatement cost ranges and technology readiness levels are informed by the broader literature on industrial decarbonisation and plastic recycling technology assessment [75,119,120]. Emission reduction potentials are estimated based on typical performance data for each technology category. TRL definitions follow standard innovation readiness frameworks.

**Table 9:** Ontology coverage summary.

Domain	Classes	Properties	Instances (Hypothetical)
Material	42	18	15 PVC grades, 12 filler types, 8 stabiliser classes
Process	28	16	6 unit operations, 3 recycling routes
Quality	24	12	18 property measurements
Sustainability	26	14	14 impact categories
Economic	18	12	8 cost types, 4 value streams
Decision Context	18	12	6 decision types (micro, macro, strategic)
Total	156	84	(variable)

**Table 10:** Computed indicators for three illustrative recycling scenarios.

Indicator	Scenario A: Mechanical (Clean Post-Industrial)	Scenario B: Mechanical (Mixed Post-Consumer)	Scenario C: Solvent-Based (Contaminated)
Yield (%)	92	78	88
Energy intensity (kWh/t)	520	680	1050
Carbon footprint (kg CO_2_e/t)	280	420	680
Operating cost (€/t)	440	560	780
Recyclate quality (grade)	Premium	Standard	Premium
Circular material use (%)	94	82	90
Abatement cost (€/t CO_2_e)	−45 (saving)	−20 (saving)	85 (cost)

**Table 11:** Net emissions by recycling route under uncertainty (kg CO_2_e/t; 100,000 Monte Carlo draws).

Recycling Route	Interval Range (Table 6)	5th Percentile	Median	95th Percentile
Mechanical (closed-loop)	−2000 to −1400	−1910	−1700	−1490
Mechanical (open-loop to lower grade)	−1000 to −400	−911	−700	−490
Solvent-based	−1500 to −800	−1390	−1150	−911
Pyrolysis (with energy recovery)	−800 to 0	−672	−400	−126
Pyrolysis (to monomers)	−1000 to −100	−859	−549	−240

Note: Process and avoided emissions were sampled independently and uniformly over the ranges of Table 6. Negative values denote net savings relative to virgin production.

**Table 12:** Operating cost (OPEX) by route under uncertainty and probability of cost recovery (€/t).

Route	Arithmetic Range (Table 5)	5th Percentile	Median	95th Percentile	Cost Recovered at €550/t	at €700/t	at €800/t
Mechanical	350–620	422	485	547	96%	100%	100%
Solvent	560–980	659	770	880	0%	17%	65%
Pyrolysis	470–900	574	686	796	2%	57%	96%

Note: Cost categories were sampled independently and uniformly over the ranges of Table 5. Cost recovery is the share of draws in which OPEX does not exceed the recyclate price; the price levels are sensitivity values and not market data.

**Table 13:** Attainment of the 2030 targets as a function of the realised share (φ) of the projected improvement.

Metric	Baseline	Projected 2030	Target	Required φ	Lower Limit of φ = 0.3	0.5	0.7
CMUR (%)	18	28	24	0.60	84%	97%	100%
EOL-RR (%)	42	58	55	0.81	53%	67%	89%
Emissions (kg CO_2_e/t), 5th–95th percentile	510	290	-	-	282–407	280–373	278–339

Note: For CMUR and EOL-RR, the last three columns give the probability that the target is met; for emissions, they give the 5th–95th percentile of 2030 emissions. φ ~ Triangular (lower limit, 1.0, 1.1) is an assumption.

**Table 14:** Provenance of the principal decision thresholds and sensitivity of the scenario decisions. **Colour notation:** (\Delta E^*) denotes the overall colour difference used to assess discolouration of the recycled PVC-u. Lower values indicate a smaller colour difference, whereas higher values indicate greater discolouration. The quality-grade thresholds are illustrative. The colour-difference formula and measurement method should be specified in the methodology.

Threshold	Value Used	Basis	Effect of Shifting the Threshold
Polyolefin flotation trigger	>2 wt% (Section 5.4; harmonised in Section 9.4)	Illustrative practitioner heuristic; to be calibrated	R01 (3 wt%) and Scenario B (4 wt%) keep the flotation decision for any threshold below 3 and 4 wt%, respectively
Lead management trigger	≥0.1 wt%; derogation ceiling 1.5 wt%	Regulation (EU) 2023/923 [21]	R01 (0.5 wt%) lies inside the band; the decision changes only if lead exceeds 1.5 wt%
Low_Grade contamination limit	>5 wt%	Illustrative	Scenario B (5 wt%) sits on the boundary; a small shift in the threshold or a measurement error changes the grade
Yield trigger	<80% (Section 7.3)	Illustrative; plant target > 85% (Section 7.1)	Scenario B (78%; 71% in the excursion) triggers; a threshold below 78% would not
Energy-intensity trigger	>800 kWh/t (Section 7.1)	Plant-level target; illustrative	Scenario B (680 kWh/t) does not trigger; Scenario C (1050 kWh/t) and the +22% excursion do
Quality-grade bands (Table 7)	ΔE* 5/10/15; K-value retention 95/85/70%	Illustrative; informed by EN 18064-5 [109] and industry practice [24,54,55]	R02 (ΔE* = 7.2) lies mid-band in Standard; robustness to noise is given in Table 15
Carbon price for the abatement test	€80/t	Observed EU ETS price of about €83/t [118]	Chemical recycling (€80–150/t) and Scenario C (€85/t) are marginal; see the abatement analysis above
Deployment priority rule	TRL ≥ 7 and reduction ≥ 70%	Illustrative	Not used in the scenario decisions

**Table 15:** Share of noisy draws that reproduce the noise-free rule decision, by relative measurement noise (coefficient of variation). **Colour notation:** (\Delta E^*) denotes the overall colour difference used to assess discolouration of the recycled PVC-u. Lower values indicate a smaller colour difference, whereas higher values indicate greater discolouration. The quality-grade thresholds are illustrative. The colour-difference formula and measurement method should be specified in the methodology.

Decision (Noise-Free Outcome)	Margin to Threshold	CV 5%	CV 10%	CV 20%	CV 30%
R01: flotation triggered (polyolefin 3 wt% vs. 2 wt%)	+50%	100%	100%	95%	87%
R01: lead within 0.1–1.5 wt% band (0.5 wt%)	inside band	100%	100%	100%	100%
R02: Standard grade retained (ΔE* = 7.2 in 5–10)	2.2 above/2.8 below	100%	100%	91%	75%
Scenario B: flotation triggered (4 wt% vs. 2 wt%)	+100%	100%	100%	99%	95%
Scenario B: yield trigger (78% vs. <80%)	−2.5%	70%	60%	55%	53%
Scenario B: not Low_Grade (5 wt% vs. >5 wt%)	0%	50%	50%	50%	50%

Note: 100,000 draws per cell. Noise is multiplicative and Gaussian and is an assumed test level, not a measured sensor specification.

## Data Availability

The original contributions presented in this study are included in the article. Further inquiries can be directed to the corresponding authors.
